# Distinguishing prognostic and predictive biomarkers: an information theoretic approach

**DOI:** 10.1093/bioinformatics/bty357

**Published:** 2018-05-02

**Authors:** Konstantinos Sechidis, Konstantinos Papangelou, Paul D Metcalfe, David Svensson, James Weatherall, Gavin Brown

**Affiliations:** 1 School of Computer Science, University of Manchester, Manchester, UK; 2 Advanced Analytics Centre, Global Medicines Development, AstraZeneca, Cambridge, UK

## Abstract

**Motivation:**

The identification of biomarkers to support decision-making is central to personalized medicine, in both clinical and research scenarios. The challenge can be seen in two halves: identifying *predictive* markers, which guide the development/use of tailored therapies; and identifying *prognostic* markers, which guide other aspects of care and clinical trial planning, i.e. prognostic markers can be considered as covariates for stratification. Mistakenly assuming a biomarker to be predictive, when it is in fact largely prognostic (and vice-versa) is highly undesirable, and can result in financial, ethical and personal consequences. We present a framework for data-driven ranking of biomarkers on their prognostic/predictive strength, using a novel information theoretic method. This approach provides a natural algebra to discuss and quantify the individual predictive and prognostic strength, in a self-consistent mathematical framework.

**Results:**

Our contribution is a novel procedure, *INFO+*, which naturally distinguishes the prognostic versus predictive role of each biomarker and handles higher order interactions. In a comprehensive empirical evaluation *INFO+ *outperforms more complex methods, most notably when noise factors dominate, and biomarkers are likely to be falsely identified as predictive, when in fact they are just strongly prognostic. Furthermore, we show that our methods can be 1–3 orders of magnitude faster than competitors, making it useful for biomarker discovery in ‘big data’ scenarios. Finally, we apply our methods to identify predictive biomarkers on two real clinical trials, and introduce a new graphical representation that provides greater insight into the prognostic and predictive strength of each biomarker.

**Availability and implementation:**

R implementations of the suggested methods are available at https://github.com/sechidis.

**Supplementary information:**

Supplementary data are available at *Bioinformatics* online.

## 1 Introduction

A *prognostic biomarker* is a clinical or biological characteristic that provides information on the likely patient health outcome (e.g. disease recurrence) *irrespective of the treatment*. On the other hand, a *predictive biomarker* indicates the likely *benefit* to the patient from the treatment, compared to their condition at baseline (Ruberg and Shen, 2015).

The difference between these two types of marker is clearly important, yet, surprisingly it is often not recognized. Ballman (2015) states that there *‘is considerable confusion about the distinction between a predictive biomarker and a prognostic biomarker.’* A specific example is highlighted by Clark (2008) when examining clinical biomarkers used routinely to make treatment decisions for non-small cell lung cancer, such as gender and histology—the key finding is that:

*‘… gender and histology are actually prognostic, rather than predictive factors. Before biomarkers or clinical characteristics are included in guidelines for selecting patients for specific treatments, it is imperative that the prognostic effects of these factors are distinguished from their ability to predict a differential clinical benefit from the specific treatment.’*

In reality, biomarkers will almost always have *some degree* of prognostic value, and *some degree* of predictive value—but will also likely be dominated by one or the other. Using a biomarker for treatment assignments (i.e. in a predictive context), when in fact it provides mostly a prognostic signal, can have personal, financial and ethical consequences—the inverse holds with different, though equally valid, consequences. A prognostic biomarker that is incorrectly labelled as predictive may result in overestimating the benefits of the treatment for a subset of the population and prescribing it to specific patients while in fact it should be available to all. Consequently, this may force the price of the drug up, as it is now considered as a treatment tailored to a specific portion of the population. The opposite applies if a predictive biomarker is incorrectly labelled as prognostic. In this case, the differential effect of the treatment to subsets of the population will be missed. The drug may be wrongly considered to have the same effect in all patients, affecting its price accordingly.

The challenge of finding markers with prognostic character is explored extensively in biostatistical and Machine Learning literature alike (Saeys *et al.*, 2007). On the other hand, discovery of *predictive* biomarkers has seen much less attention in Machine Learning, e.g. Su *et al.* (2008). The biomedical literature on *subgroup identification* (Ondra *et al.*, 2016) includes predictive biomarker ranking as an intermediate step, with SIDES (Lipkovich *et al.*, 2011), Virtual Twins (Foster *et al.*, 2011) and Interaction Trees (Su *et al.*, 2009) as recent examples in this direction. However, little attention has been paid to the challenge of explicitly *distinguishing* between markers with mixed predictive/prognostic value. A statistical tool that could explicitly distinguish and *quantify* the *predictiveness* and *prognosticness* of a biomarker may be useful in study design and clinical interpretation of predictive models. In our work, we propose a unified approach that provides a language highly suited to biomarker discovery and related tasks around personalized medicine.

Our strategy is to align the challenges of data-driven biomarker discovery with that of *information theoretic* feature selection (Brown *et al.*, 2012). The information theoretic viewpoint provides an ‘algebra’ with well-defined semantics, enabling one to discuss and quantify the predictiveness/prognosticness of a biomarker—each separately, but in a self-consistent mathematical framework. This also formalizes the problem as finding the biomarkers that optimize a well-defined objective function, capturing the *joint* influence of patient characteristics and the treatment regime, on the outcome. Stated informally, this is:

$$\begin{array}{c} \text{Joint}\;\text{effect}\;\text{of}\;\text{patient}\;\text{characteristics}\\ \text{and}\;\text{treatment}\;\text{on}\;\text{outcome} \end{array}=\begin{array}{c} \text{Prognostic}\\ \text{effect} \end{array}+\begin{array}{c} \text{Predictive}\\ \text{effect} \end{array}$$

We will show how this plain English statement can be formalized with the algebra of information theory—and allow us to *rank* biomarkers by their prognostic or predictive value strength, as measured in ‘bits’ of information (Shannon, 1948). It is also important to remember that univariate rankings have limitations—Lipkovich *et al.* (2017) recently stated that

*‘…the key deficiency of univariate* [.] *models is that they ignore potential synergistic effects of two or more biomarkers by failing to account for higher-order interaction effects.’*

To deal with this problem, methods such as Interaction Trees and SIDES take a strategy of *recursively* partitioning the data, isolating regions of the space of patients as functions of two or more biomarkers. However, this results in small-sample issues, and hyper-parameters for model-building in different data partitions. Using the information theoretic approach, we derive a novel method, *INFO+*, that captures second-order biomarker interactions, and comes with natural solutions to the small-sample issue.

We evaluate the performance of the competing methods with an extensive experimental comparison, to highlight their strengths and weaknesses in identifying predictive markers. We systematically increase the challenge in simulated data—for example: having biomarkers that are solely predictive, or of mixed predictive/prognostic value, having correlated biomarkers, or having an enhanced predictive signal in a subgroup of patients. We will demonstrate that *INFO+ *empirically outperforms competing methods, not only in true positive/negative rates of different marker types, but also in terms of computational- and data-efficiency. Finally, we introduce a visualization tool for the prognosticness and the predictiveness of a set of biomarkers. We hope that the proposed visualization method will become a standard in the practitioners’ toolkit for identifying important biomarkers and understanding their effects.

## 2 Materials and methods

In this section we build links between data-driven biomarker discovery and *information theoretic feature selection* (Brown *et al.*, 2012). Firstly, we formally define the concept of predictive versus prognostic biomarkers, in a language familiar to the bioinformatics community; following this, we provide an *information theoretic* formalization, leading to a set of novel methods for predictive biomarker ranking.

### 2.1 Prognostic versus predictive biomarkers

The distinction between predictive and prognostic markers can be ambiguous if expressed in natural language. However, it becomes simple to illustrate and agree upon, if we assume a known underlying model generating the data. We will use this tactic below, *using a linear model as a purely illustrative tool*—though all novel methods in the paper will apply in the general case. We denote the health outcome *Y* as a function of the patient characteristics X and the treatment *T*, for example:

$$f(X,T)=\alpha_{1}X_{1}+\alpha_{2}X_{2}+(\beta_{2}X_{2}+\beta_{3}X_{3})T,$$

where *α*_1_ and *α*_2_ coefficients define the prognostic elements, and *β*_2_ and *β*_3_ define the predictive. When *Y* is continuous, f(X,T) can model the conditional mean E(Y|X,T), alternatively, if *Y* is binary, f(X,T) can be the logit of the conditional probability: logit[P(Y=1|X,T)]. If data is generated according to the model above, *X*_1_ is regarded as solely *prognostic* as it *directly* influences the outcome *Y*—also known as a *main effect*. The variable *X*_3_ is solely *predictive* as it influences the outcome only via an interaction with the treatment variable—also known as an *interaction effect*. However, note that variable *X*_2_ has *both predictive and prognostic* strength.

Following standard notation (Lipkovich *et al.*, 2017), assuming linear interactions, the outcome function can be written as:

f(X,T)=h(X)+z(X)T,

where h(.) and z(.) are arbitrary functions of the covariates. We stress again that this is for the purposes of exposition, and all novel methods/results in the paper apply without such assumptions. There are two challenges here—firstly ranking variables in X by how much they influence *Y* via h(X) (i.e. main effects)—we refer to this as **prognostic ranking**. And, secondly, ranking those that influence *Y* via z(X) (i.e. interaction effects)—we refer to this as **predictive ranking**.

Assuming a binary treatment T∈{0,1}, simple algebra yields that *z* is the change in outcome *Y* due to treatment, known as *treatment effect*:

z(X)=f(X,1)−f(X,0).

The estimation of treatment effect (via first estimating f(X,·)) motivates a set of methods that utilize the potential outcomes (or counterfactual) modelling (Rubin, 1974). For example, a popular method in this class is Virtual Twins (VT) (Foster *et al.*, 2011), which non-parametrically estimates $\hat{f}(X,T)$ using random forests (RF) (Breiman, 2001). Predictive rankings can then derived by ranking the biomarkers on their dependence with the *estimated* variable z(X). Another class of methods for predictive ranking is based on recursively partitioning data using interaction tests between treatment and covariate—two popular methods here are SIDES (Lipkovich *et al.*, 2011) and Interaction Trees (Su *et al.*, 2009). Section S1 of Supplementary Material provides details on all of the competing methods.

We take a different approach, by reformulating the problem as an optimization of an information theoretic objective. We introduce a new method for deriving predictive rankings, without the assumption of linear models, or binary *T* as above. Our method is directly applicable to multi-arm trials (i.e. more than two treatment groups) and captures higher-order biomarker interactions. In the following sections we introduce our framework.

### 2.2 A natural objective function

Shannon (1948) in his seminal work ‘A Mathematical Theory of Communication’ introduced information theory to quantify the amount of information and the capacity of the communication channel. One of the most fundamental concepts is *mutual information*. Through time, information theoretic approaches based on mutual information used to solve challenging problems in various research areas, e.g. Physics (Lloyd, 1989), Bioinformatics (Steuer *et al.*, 2002) and Machine Learning (Zeng, 2015).

In simple terms, the mutual information I(X;Y) captures the extent to which two random variables *X*, *Y* depend on each other, or in other words the reduction of uncertainty in one variable *Y* given the values of the other *X*. Mutual information has various interesting properties. For example, it can be associated with both upper and lower bounds on the Bayes error (Zhao *et al.*, 2013). As a result, optimizing information theoretic measures to solve challenging problems, i.e. feature selection (Brown *et al.*, 2012), can lead to methods with competitive performance.

In clinical trial data, a natural way to select a set of biomarkers is to *maximise the shared mutual information between the target Y and the joint random variable of the treatment T and the optimal feature set*$X^{*}$. Using the same analysis as in (Brown *et al.*, 2012) it can be proved that this criterion is equivalent to *maximise the conditional likelihood of the outcome given the features and treatment*, i.e. log ⁡p(Y|XT). Using the mutual information chain rule (Cover and Thomas, 2006), this objective can be decomposed in the following way:

$$X^{*}=\underset{{}_{X_{\theta }\in X}}{\text{arg max}}I(X_{\theta }T;Y)=\underset{{}_{X_{\theta }\in X}}{\text{arg max}}(\underset{\text{Prognostic part}}{\underset{︸}{I(X_{\theta };Y)}}+\underset{\text{Predictive part}}{\underset{︸}{I(T;Y|X_{\theta })}})$$

The first part captures the biomarkers with prognostic strength, while the second captures the biomarkers with predictive strength (Section S2 of Supplementary Material provides some background on information theory, while Section S3 motivates why these two terms capture the prognostic and predictive strength by connecting the estimation of the mutual information with the deviance of the log-linear models.). By optimizing these two parts *independently* we can derive two different objectives for the two different biomarker sets:

(1) $$X_{\text{Prog}.}^{*}=\underset{{}_{X_{\theta }\in X}}{\text{argmax}}\;I(X_{\theta };Y),\;$$

(2) $$X_{\text{Pred}.}^{*}=\underset{{}_{X_{\theta }\in X}}{\text{argmax}}\;I(T;Y|X_{\theta }).$$

Brown *et al.* (2012), in the context of feature selection, presented two heuristics for optimizing Eq. (1), which consider sequentially features one-by-one for adding or removal; the forward selection and the backward elimination respectively. For example, in forward selection at each step *k* we select the feature $X_{k}^{*}$ that maximizes the conditional mutual information (CMI):

(3) $$X_{k}^{*}=\underset{{}_{X_{k}\in X_{\tilde{\theta }}}}{\text{arg max}}J_{\text{CMI}}(X_{k})=\underset{{}_{X_{k}\in X_{\tilde{\theta }}}}{\text{arg max}}I(X_{k};Y|X_{\theta })$$

where $X_{\theta }$ is the set of the features already selected, and $X_{\tilde{\theta }}$ the unselected. The CMI criterion can be used to rank the biomarkers on their prognostic strength and it is provably a hill-climber on the likelihood (Brown *et al.*, 2012). In the following section we extend this result to derive predictive biomarker rankings.

### 2.3 Information theoretic predictive rankings

In order to rank the biomarkers on their predictive strength, we should derive an optimization procedure for the predictive part Eq. (2). A common heuristic approach to optimize an objective like this is to sequentially consider biomarkers one-by-one for adding or removal. This heuristic can be seen as a greedy iterative optimization of the desired objective function. To present this procedure we will use a time-index notation for the feature sets, where $X_{\theta^{\tau }}$ represent the selected features at timestep *τ*, while $X_{{\tilde{\theta }}^{\tau }}$ the unselected ones.

**Definition 1** (Predictive biomarker forward selection step with CMI). The forward selection step adds the biomarker $X_{k}^{*}$ which maximizes the conditional mutual information between *T* and *Y* in the context of the joint variable of the currently selected set $X_{\theta^{\tau }}$ and $X_{k}^{*}$. The operations performed are:

$$\begin{array}{l} X_{k}^{*}=\underset{{}_{X_{k}\in X_{{\tilde{\theta }}^{\tau }}}}{\text{arg max}}I(T;Y|X_{\theta^{\tau }}X_{k})\\ X_{\theta^{\tau +1}}\leftarrow X_{\theta^{\tau }}\cup X_{k}^{*}\\ X_{{\tilde{\theta }}^{\tau +1}}\leftarrow X_{{\tilde{\theta }}^{\tau }}\backslash X_{k}^{*} \end{array}$$

Using our formalization of the problem and the results of Brown *et al.* (2012) the following theorem holds.

**Theorem 1.** The predictive forward selection heuristic adds the biomarker that causes the largest increase in the predictive part.

While for the backward elimination we have the following definition:

**Definition 2.** (Predictive biomarker backward elimination step with CMI). The backward elimination step removes the biomarker $X_{k}^{*}$ which minimizes the conditional mutual information between *T* and *Y* in the context of the variable of the currently selected set $X_{\theta^{\tau }}$ without $X_{k}^{*}$. The operations performed are:

$$\begin{array}{l} X_{k}^{*}=\underset{{}_{X_{k}\in X_{\theta^{\tau }}}}{\text{arg min}}I(T;Y|\{X_{\theta^{\tau }}\backslash X_{k}\})\\ X_{\theta^{\tau +1}}\leftarrow X_{\theta^{\tau }}\backslash X_{k}^{*}\\ X_{{\tilde{\theta }}^{\tau +1}}\leftarrow X_{{\tilde{\theta }}^{\tau }}\cup X_{k}^{*} \end{array}$$

Using the results of Brown *et al.* (2012) the following theorem holds.

**Theorem 2.** The predictive backward elimination heuristic removes the marker that causes the minimum possible decrease in the predictive part.

For simplicity from now on we will focus on the forward selection procedure, where at each step we select the feature not ranked so far $X_{k}^{*}\in X_{\tilde{\theta }}$ that maximizes the following score:

(4) $$X_{k}^{*}=\underset{{}_{X_{k}\in X_{\tilde{\theta }}}}{\text{arg max}}J_{\text{PRED-CMI}}(X_{k})=\underset{{}_{X_{k}\in X_{\tilde{\theta }}}}{\text{arg max}}I(T;Y|X_{\theta }X_{k})$$

We will call this criterion as PRED-CMI, since it measures the predictive strength of each biomarker *X_k_* by measuring the conditional mutual information between treatment and outcome given the joint variable between *X_k_* and the currently ranked biomarkers $X_{\theta }$. In the following section we propose low-dimensional approximations, that will allow us to reliably estimate the conditional mutual information when the dimensionality of $X_{\theta }$ becomes prohibitively high.

### 2.4 Lower-dimensional criteria for predictive rankings

In both scenarios, deriving prognostic rankings using CMI, and deriving predictive rankings using PRED-CMI, we need to tackle an important challenge: as the number of selected features grows, the dimension of $X_{\theta }$ also grows, and this makes our estimations less reliable. To overcome this problem *low-dimensional* criteria need to be derived.

For deriving prognostic rankings, the machine learning literature for feature selection is vast of low-order criteria. Brown *et al.* (2012) showed that a criterion that controls relevancy, captures feature interactions through redundancy and complementarity and provides a very good tradeoff in terms of accuracy, stability and flexibility is the *Joint Mutual Information* (JMI) criterion (Yang and Moody, 1999): $J_{\text{JMI}}(X_{k})=\sum_{X_{j}\in X_{\theta }}I(X_{k};Y|X_{j}).$

Now we will tackle the challenge of deriving low-order criteria for the predictive rankings. The simplest way is to measure the conditional mutual information of *T* and *Y* given each biomarker *independently* without any regards of the others. This criterion can be seen as a *univariate* information theoretic way to derive predictive rankings, and from now on we will call it *INFO*. The score that *INFO* uses to rank the biomarkers is:

(5) $$J_{INFO}(X_{k})=I(T;Y|X_{k}).$$

While this is a low-dimensional criterion—we simply need to estimate the conditional mutual information between three variables—it fails to capture the dependencies between the biomarkers, i.e. collinearity/multicollinearity. We can illustrate these dependencies better by using the information theoretic identity I(A;B|CD)=I(A;B|D)−I(C;B|D)+I(C;B|AD) to re-write the PRED-CMI criterion as

(6) $$J_{\text{PRED}-\text{CMI}}(X_{k})=I(T;Y|X_{k})-I(X_{\theta };Y|X_{k})+I(X_{\theta };Y|TX_{k}).$$

The first term captures the predictive strength of the biomarker *X_k_* being considered for inclusion in $X_{\theta }.$ The second term capture the three-way interaction between the existing biomarker set $X_{\theta }$, the target *Y* and the biomarker *X_k_*. Finally, third term captures the four-way interaction between $X_{\theta }$, *Y*, *T* and the biomarker $X_{k}.$ The last two are those that capture the collinearity between *X_k_* and $X_{\theta }$. We refer to these three terms as: the *predictive relevancy*, the *predictive redundancy* and the *predictive complementarity* of the biomarkers.

As we see, *INFO* is an approximation of PRED-CMI that captures only the first term, which measures the predictive strength of biomarker *X_k_*, but it fails to account for terms that capture the redundancy between the biomarkers. These, the predictive redundancy and predictive complementarity, are high dimensional functions of $X_{\theta }$—we can approximate them (to second-order interactions) with a sum as follows.

(7) $$J_{INFO+}(X_{k})=\sum_{X_{j}\in X_{\theta }}I(T;Y|X_{j}X_{k}).$$

We refer to this as *INFO+*, and using the same identity as before, it can be re-written in the following ranking-equivalent form:

$$J_{INFO+}(X_{k})\;\propto \;I(T;Y|X_{k})-\frac{1}{\left|X_{\theta }\right|}\sum_{X_{j}\in X_{\theta }}\left[I(X_{j};Y|X_{k})-I(X_{j};Y|TX_{k})\right],$$

where $\left|X_{\theta }\right|$ is the number of biomarkers already ranked. As we see from the last expression, *INFO+ *captures all the three desirable terms for predictive biomarker ranking: predictive relevancy, predictive redundancy, and predictive complementarity. Furthermore, as the size of $X_{\theta }$ grows, *INFO+ *does not involve high dimensional conditional information estimations as the ones in PRED-CMI Eq. (6). Instead it averages over all possible lower dimensional (pairwise) terms, $I(X_{j};Y|X_{k})$ and $I(X_{j};Y|TX_{k})\;\;\;\forall \;X_{j}\in X_{\theta }$. By averaging over all possible pairwise terms, *INFO+ *captures second-order biomarker interactions. In theory this could be extended to arbitrary higher order interactions, but data constraints will always limit this.

Algorithm 1 describes our approach for deriving predictive biomarker rankings. The provided algorithm is in a user-friendly form for illustrative purposes, but can easily be optimized to be 2–3 orders of magnitude faster than a direct translation. Note that in some scenarios i.e. subgroup identification (Foster *et al.*, 2011), and especially in high-dimensional settings, we might be interested in only a few biomarkers (Lipkovich and Dmitrienko, 2014). To this end, in contrast to existing methods (i.e. SIDES/VT/IT) which rank all biomarkers, our *INFO+ *forward step-wise procedure can return only the top-*K*, without the need to rank all of them. This capability removes a significant computational burden (see Section 3.1.10).

As we already mentioned, our methods rank the biomarkers by estimating conditional mutual information quantities. To this end we can use any off-the-shelf estimator suggested in the literature (Section S4 of the Supplementary Material provides a short review and details on how to estimate conditional mutual information.). Since in clinical trials we often encounter small-samples, in our implementation we used a shrinkage estimator suitable for ‘small *n*, large *p*’ scenarios (Hausser and Strimmer, 2009). This approach can be extended to handle various types of covariates, i.e. categorical, continuous and mixed and various types of outcomes, i.e. categorical, continuous and survival. Furthermore, when we have mixed type of data direct comparison of the mutual information values might be problematic. To overcome this issue, we use normalized versions of the conditional mutual information, which take into account the diverse characteristics of each covariate (Vinh *et al.*, 2010).

Algorithm 1 Greedy forward selection for *INFO+* ranking

**Input:** Clinical trial data X,T,Y and size of the returned ranking *K*

**Output:** List of top-*K* predictive biomarkers $X_{\theta }$

1: $X_{\tilde{\theta }}=X$            ▹ Set of candidate biomarkers

2: Set $X_{\theta }$ to empty list      ▹ List of selected biomarkers

3: **for** k:= 1 to K **do**

4:  Let $X_{k}^{*}\in X_{\tilde{\theta }}$ maximise $J_{INFO+}(X_{k})=\sum_{X_{j}\in X_{\theta }}I(T;Y|X_{j}X_{k})$

5:  $X_{\theta }(k)=X_{k}^{*}$        ▹ Add biomarker $X_{k}^{*}$ to the list

6:  $X_{\tilde{\theta }}=X_{\tilde{\theta }}\backslash X_{k}^{*}$ ▹ Remove biomarker $X_{k}^{*}$ from the candidate set

7: **end for**

## 3 Results

This section presents a comprehensive study in comparing our information theoretic methods with state-of-the-art approaches for biomarker rankings that capture their predictive strength. We will compare our methods (INFO and INFO+) against various methods, which can be discussed/characterized from the perspective of the statistical tools each is using: penalized linear regression methods [such as MCR (Tian *et al.*, 2014)], counterfactual modelling methods [such as VT (Foster *et al.*, 2011)] and recursive partitioning methods [such as SIDES (Lipkovich *et al.*, 2011) and IT (Su *et al.*, 2008)]. Section S5 of the Supplementary Material provides the necessary details regarding the implementation of the competing methods.

### 3.1 Experiments with simulated data

We simulate a large number of different scenarios and Section 3.1.1 presents all the necessary details of the simulation models. Section 3.1.2 presents the evaluation measures that we will use. Finally, Sections 3.1.3–3.1.10 explore empirically a series of interesting questions for the performance characteristics of the different methods.

#### 3.1.1 Simulation models

With our simulated models we capture a wide variety of different scenarios. For each model we simulate data with various size *n* and dimensionality *p*. For each dataset we assumed equal allocations of patients to intervention and placebo arms, i.e. a clinical trial with 1:1 randomization. Following Lipkovich *et al.* (2011) experimental setting, most of our models emulate the challenging scenario of ‘failed’ clinical trials, where the overall treatment effect in a population is nonexistent. We simulated data using different logistic regression models, categorized in three levels of difficulty: ‘easy’, ‘medium’ and ‘hard’ with the different functional forms f(X,T)=logit[P(Y=1|T,X)]. Section S6 of Supplementary Material presents in detail the simulation models. Table 1 summarizes all the above models in increasing challenge for identifying predictive biomarkers. In the experiments of the main paper we focused on categorical covariates, so in all scenarios, after we generated the data, each covariate was discretized in 2–5 levels using an equal-width strategy (Section S7 of Supplementary Material presents experimental results using continuous covariates). Furthermore, in order to have a better control over the effect of the prognostic (i.e. main-effects) and predictive part (i.e. interaction-effects), for each part we use the same functional form but with different variables. By following this approach we can control the relative strength of the predictive part using a coefficient θ.

**Table 1.** Different scenarios of increasing challenge in identifying predictive biomarkers

	Fully separate pred/prog biomarkers?	Correlated biomarkers?	Interaction terms?	Subgroups?
M-1				
M-2	*✓*			
M-3	*✓*	*✓*		
M-4, M-5	*✓*	*✓*	*✓*	
M-6, M-7	*✓*	*✓*	*✓*	*✓*

*Notes*: **Fully-separate pred/prog biomarkers** is where there are no biomarkers with *both* predictive and prognostic strength, so a method cannot find a predictive biomarker by simply picking up on its prognostic nature. **Correlated covariates** creates situations where we might mistakenly pick up a noisy/prognostic biomarker, as it may be correlated to the predictive one for which we are searching. **Interaction terms** creates situations where two biomarkers interact to cause the outcome, which needs to be accounted for in the biomarker discovery algorithm. The presence of **Subgroups** creates situations where clearly defined groups of patients have enhanced treatment effect. More ticks equate to a more challenging scenarios.

#### 3.1.2 Evaluation measures

One natural evaluation measure is to check how accurate are the different methods on correctly placing the predictive biomarkers in the top of the rankings. Let us define as $X_{\text{Pred}.}$ the set of biomarkers with predictive strength, with size $q=|X_{\text{Pred}.}|$. Also, ${\hat{X}}_{\text{Pred}.}^{q}$ the set of the top-*q* biomarkers returned by any of the methods that produce predictive rankings. We define as true positive rate (TPR) the fraction of predictive biomarkers correctly ranked in the top-*q* positions of the list:

$$\text{TPR}=\frac{\left|X_{\text{Pred}.}\cap {\hat{X}}_{\text{Pred}.}^{q}\right|}{q}$$

Since our main objective is to introduce an information theoretic method for disentangling predictive and prognostic strength, it will be interesting to see how many times prognostic biomarkers are mistakenly placed in the top of the predictive rankings. Let us define as $X_{\text{Prog}.}\backslash X_{\text{Pred}.}$ the set of markers with *solely prognostic* strength, while $X_{\text{Irr}.}$ are the irrelevant markers, i.e. the ones that they are neither prognostic nor predictive. The false negative rate (FNR = 1 − TPR) can be decomposed in two terms:

$$\text{FNR}=\frac{\left|\left(X_{\text{Prog}.}\backslash X_{\text{Pred}.}\right)\cap {\hat{X}}_{\text{Pred}.}^{q}\right|}{q}+\frac{\left|X_{\text{Irr}.}\cap {\hat{X}}_{\text{Pred}.}^{q}\right|}{q}={\text{FNR}}_{\text{Prog}.}+{\text{FNR}}_{\text{Irr}.}$$

TPR captures how accurate are the algorithms in correctly identifying the predictive biomarkers, while ${\text{FNR}}_{\text{Prog}.}$ how often they tend to select as predictive biomarkers those that contain only prognostic information.

We generate test data from the simulation models, and rank the biomarkers on their predictive strength using the methods presented above. In this synthetic situation, we have the ground truth set of biomarkers that have any degree of predictive strength: $X_{\text{Pred}.}$, and the same for prognostic markers $X_{\text{Prog}.}.$ We use this to calculate the TPR and ${\text{FNR}}_{\text{Prog}.},$ for each ranking. Finally, we report the average results over multiple simulated datasets. For the ranking methods that use an estimate of the classification error to produce a variable importance score, such as VT, and in order to avoid overfitting, we use out-of-bag estimates (Foster *et al.*, 2011).

#### 3.1.3 Does markers’ prognostic strength affect the predictive ranking?

**Fig. 1.**
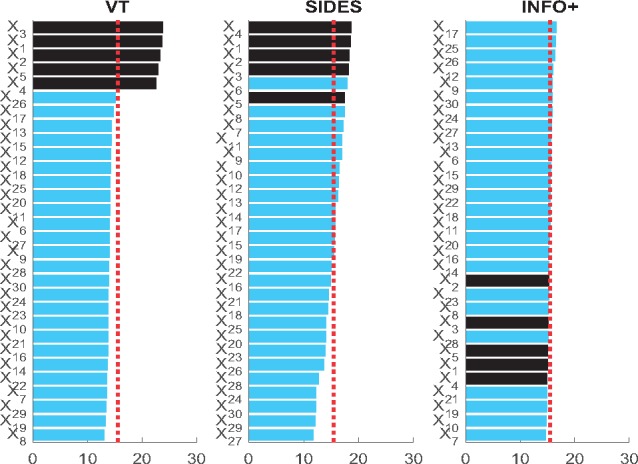
VT and SIDES, whilst searching for predictive signals, mistakenly give high rank to variables that are purely prognostic, with no predictive signal whatsoever (black bars); whereas, INFO+ correctly assigns them a rank no better than random. This is the average ranking score over 200 simulated datasets generated by model **M-1**, in the absence of any predictive information *θ* = 0, sample size 2000 and dimensionality *p* = 30 biomarkers. The dashed line is the average expected score, representing a ranking by random chance. Anything above can be considered as significant

Before exploring in depth the performance of the different methods for deriving predictive biomarkers, the first thing we should explore is whether the ranking they produce is biased towards the prognostic strength of each biomarker. To answer this question we generate 200 datasets from the **M-1** model with *p* = 30 biomarkers and *without* any predictive information, i.e. setting *θ* = 0. Under this model, we have *no predictive biomarker*, five prognostic $X_{1},\ldots X_{5}$, and the rest are irrelevant. To rank the biomarkers on their predictive strength we use three different methods (*INFO+*, VT, SIDES), and we derive the ranking score as follows: the most important marker takes score 30, the second most important 29 till the least important which takes score 1. Since there is no predictive biomarker, we expect that on average the score of each biomarker should be the same, ≈15.5. Figure 1 shows that VT is biased towards the prognostic biomarkers, i.e. $X_{1},\ldots X_{5}$, since on average, these biomarkers get higher score and they are on the top of the list. SIDES is also biased towards prognostic markers, but in smaller extent than VT. Our method, *INFO+*, is not biased towards the prognostic strength, since it produces equal scores for each biomarker. This also shows that ranking biomarkers on their conditional mutual information I(T;Y|X), captures the predictive strength, and not any prognostic information.

**Remark 1:** VT appears to show bias towards the prognostic biomarkers, whereas INFO+ is not, since the conditional mutual information, I(T;Y|X), explicitly captures the predictive strength.

#### 3.1.4 What happens when biomarkers have solely predictive strength?

**Fig. 2.**
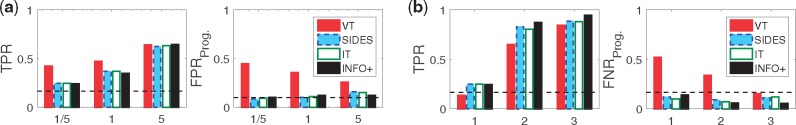
When biomarkers have both prognostic/predictive strength (M-1) VT achieves higher TPR, otherwise (M-2) the gains in TPR are vanishing. In terms of ${\text{FNR}}_{\text{Prog}.}$, VT always has very high error rate on selecting *solely* prognostic biomarkers as predictive, and it performs worse than random selection. This is the average TPR/${\text{FNR}}_{\text{Prog}.}$ over 200 simulated datasets for three different values of the predictive strength *θ*: 1/5 means a strongly prognostic signal, 1 means equal strength between prognostic and predictive signals, and 5 means a strongly predictive signal. The sample size is 2000, and the dimensionality *p* = 30 biomarkers. Dashed lines show the TPR/${\text{FNR}}_{\text{Prog}.}$ if we were ranking the biomarkers at random. (**a**) **M-1:** Biomarkers can be both prognostic and predictive. (**b**) **M-2:** Biomarkers are solely either prognostic or predictive

The experiments of this section focus on two scenarios where the predictive biomarkers have diverse nature. Firstly, when we have predictive biomarkers that carry also prognostic information (**M-1**), and, secondly, when we have models that the predictive biomarkers do not appear in the prognostic part (**M-2**). Figure 2 presents an interesting finding. The results in model **M-1** show that VT achieves very high TPR, especially for scenarios with small predictive signals (i.e. θ=1/5), but on the other hand ${\text{FNR}}_{\text{Prog}.}$ is also very high. In other words, when there is a strongly prognostic signal, VT falsely assumes prognostic biomarkers as predictive. However, in model **M-2**, when biomarkers cannot have mixed predictive/prognostic nature, TPR of VT drops dramatically, *and*${\text{FNR}}_{\text{Prog}.}$ remains high.

By comparing the results of the two models we can conclude that when we have biomarkers with both predictive and prognostic strength (i.e. **M-1**), VT achieves high TPR, but when the two sets are distinct (i.e. **M-2**) the gains in TPR are vanishing. In terms of ${\text{FNR}}_{\text{Prog}.}$, VT always has very high error rate on selecting *solely* prognostic biomarkers as predictive, and it performs always worse than random selection. This highlights that VT is somewhat biased towards the biomarkers with strong prognostic effect. Furthermore, from Figure 2 we observe that the recursive partitioning methods (SIDES/IT) perform very similar in all scenarios, while our *INFO+ *method outperforms all of the rest in almost every setting, and it achieves a better trade-off between TPR/${\text{FNR}}_{\text{Prog}.}$.

**Remark 2:** VT is biased towards predictive biomarkers that also carry prognostic information. *INFO+ *achieves better performance by disentangling the predictive and prognostic information of each biomarker.

#### 3.1.5 What happens when we have correlated and interacted markers?

In this section we motivate the necessity of multivariate methods, such as *INFO+*, that capture higher-order biomarker interactions. We will compare *INFO+ *with two univariate approaches: our information theoretic *INFO*, and MCR, which, due to the linear modelling, does not capture higher order biomarker interactions. For the purpose of this section we will focus on three models **M-2**, **M-3** and **M-4** with diverse characteristics.

**Fig. 3.**
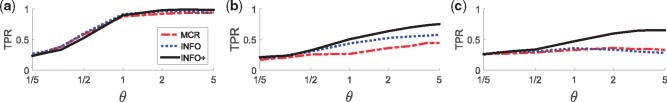
*INFO+ *captures correlations (M-3) and high-order biomarker interactions (M-4), and it outperforms methods that fail to capture these complex structures (i.e. INFO/MCR). This is the average TPR over 200 simulated datasets for various values of the predictive strength *θ*: small values of *θ* mean that the prognostic signal is stronger than the predictive, while the opposite holds for large values of *θ*. For *θ* = 1 both signals have the same strength. The sample size is 2000 and the dimensionality *p* = 30 biomarkers. (**a**) **M-2:** Uncorrelated features, no interaction terms. (**b**) **M-3:** Correlated features, no interaction terms. (**c**) **M-4:** Correlated features, with interaction terms

Model **M-2** does not contain higher order interactions and the biomarkers are uncorrelated. In this case we expect that higher-order methods do not provide any actual benefit, and Figure 3 verifies it. All three methods have similar performance in terms of TPR, and this holds for various values of the predictive strength *θ*. However, this is not the case when we have *correlated* features (model **M-3**). In this case *INFO+ *outperforms the univariate methods, and this trend is even stronger when we also have interaction terms in the model (model **M-4**). In the latter scenario the univariate methods completely fail, even with strong predictive signals.

**Remark 3:** *INFO+ *captures interactions between biomarkers without the need to explicitly model the functional form of the predictive part.

#### 3.1.6 What happens when we change the sample size?

**Fig. 4.**
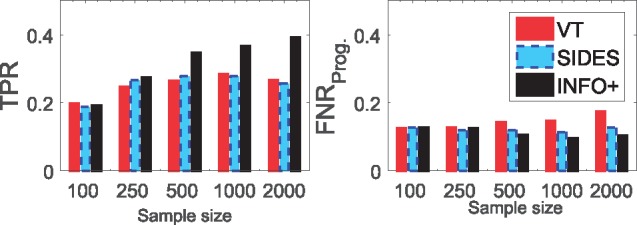
Comparing VT/SIDES/*INFO+ *for varying sample sizes. This is the average TPR/${\text{FNR}}_{\text{Prog}.}$ over 200 simulated datasets from model **M-5** with various sample sizes *n*. We simulated the data with predictive strength *θ* = 5 and dimensionality *p* = 30

In this section we compare the different methods in terms of their efficacy with the sample size. We focus on the medium difficulty model **M-5** and we explore how the different methods perform as we vary the sample size. We simulate from small trials of *n* = 100 subjects, up to larger ones with *n* = 2000. Figure 4 presents our findings. Our method achieves higher TPR, increasing faster with *n*, and similarly shows a more rapid decrease in ${\text{FNR}}_{\text{Prog}.}$, outperforming the competitors.

**Remark 4:** *INFO+ *is the most sample efficient method, i.e. it converges faster with the sample size.

#### 3.1.7 What happens when we increase dimensionality?

**Fig. 5.**
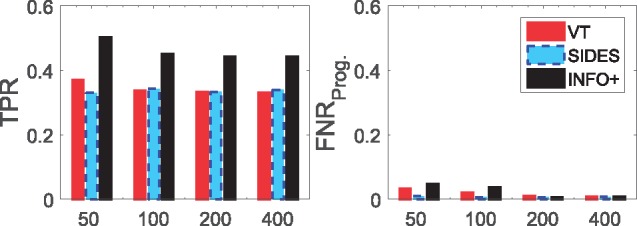
Comparing VT/SIDES/*INFO+ *for problems with different dimensionalities. This is the average TPR/${\text{FNR}}_{\text{Prog}.}$ over 200 simulated datasets from **M-6** various dimensionalities *p*. We simulated the data with predictive strength *θ* = 5 and sample size *n* = 2000

Another interesting hypothesis to explore is how the above methods perform when we have a large number of covariates/biomarkers. To explore this we use the medium difficulty model **M-6** and on Figure 5 we present how the different methods perform for various dimensionalities, p={50,100,200,400} covariates. As we see *INFO+ *consistently outperforms the other methods in terms of TPR, for both low and high dimensional trials, while it controls very well ${\text{FNR}}_{\text{Prog}.}$.

**Remark 5:** *INFO+ *is the most efficient method in the presence of large number of noisy variables.

#### 3.1.8 What happens when we have different size of subgroups?

As we already mentioned, an important usage of predictive biomarkers is to define subgroups of people with an enhanced treatment effect (Lipkovich *et al.*, 2017). Defining these subgroups is crucial for *personalised medicine*, and in this section we will explore how the methods perform, in the presence of such subgroups. We will focus on models **M-6** and **M-7**, which have subgroups with diverse characteristics.

**Fig. 6.**
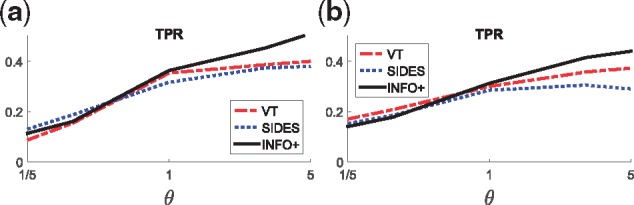
Comparing VT/SIDES/*INFO+ *for problems with subgroups with enhanced treatment effect. This is the average TPR over 200 simulated datasets for various values of the predictive strength *θ*. The sample size is 2000 and the dimensionality *p* = 30 biomarkers. (**a**) **M-6:** 50% of the examples, defined by two biomarkers, have an enhanced treatment effect. (**b**) **M-7:** 25% of the examples, defined by three biomarkers, have an enhanced treatment effect

Figure 6 shows that when we have subgroups that are defined by a small number of biomarkers, such as two in **M-6**, our method achieves better TPR than the other two. This trend is more marked in medium to strong predictive signals (i.e. θ≥1), while for weak signals all the methods perform similarly. The same holds for more complex scenarios, i.e. **M-8**, where the subgroup is defined by a three-variable interaction term.

**Remark 6:** *INFO+ *achieves competing performance in ranking biomarkers in the presence of subgroups with an enhanced treatment effect.

#### 3.1.9 What happens in trials with a significant overall treatment effect?

**Fig. 7.**
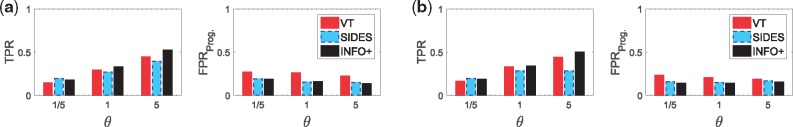
Comparing VT/SIDES/*INFO+ *for models that simulate successful trials, where there is a treatment effect on the outcome independently of the covariates. This is the average TPR over 200 simulated datasets for various values of the predictive strength *θ*: small values of *θ* mean that the prognostic signal is stronger than the predictive, while the opposite holds for large values of *θ*. For *θ* = 1 both signals have the same strength. The sample size is 2000 and the dimensionality *p* = 30 biomarkers. (**a**) **M-8:** Common treatment effect. (**b**) **M-9:** Stochastic subject-specific treatment effect

So far our models (**M-1**–**M-7**) simulated scenarios of ‘failed’ clinical trials, where the treatment effect in the population is nonexistent, and there was a significant effect only within a small subgroup of the population. In this section we will focus on the performance of the algorithms when there is a significant effect of the treatment across the whole population, i.e. a successful trial. To emulate this scenario we use two medium difficulty models **M-8** and **M-9**, with diverse characteristics: the first one has a common treatment effect for all the examples, while the second has a stochastic subject-specific treatment effect, generated independently of the covariates (Section S6 of Supplementary Material provides more details). Figure 7 presents how the different methods perform for various strengths of the predictive signal. Our method outperforms the other methods in terms of TPR, especially for medium and high predictive effects, while achieving lower ${\text{FNR}}_{\text{Prog}.}$.

**Remark 7:** *INFO+ *outperforms the competing methods when we have successful trials, i.e. when there is strong treatment effect on the outcome independently of the covariates.

#### 3.1.10 Which algorithm is more computationally efficient?

Lastly, it will be interesting to compare the performance of the methods in terms of their computational complexity. For this set of experiments we compare the average CPU time that each method needs to return the rankings, and see how it scales with the sample size and the dimensionality. All the experiments were run on a PC with Intel [textregistered] Core(TM) i5-2400 CPU @ 3.10 ghz and 8 GB RAM, on a 64-bit Windows 7 OS.

Furthermore, we will use our optimized computational implementation of *INFO+*. In the user-friendly *INFO+ *implementation presented in Alg. 1, every time we select a marker we estimate from scratch the *INFO+ *score, or in other words we need to estimate $\left|X_{\theta }\right|$ conditional mutual information terms for each unselected biomarker (Alg. 1 Line 4). But we can optimize this process by storing the score of *each* unselected biomarker, and update it in every iteration. With this optimization, instead of estimating $\left|X_{\theta }\right|$ terms for every unselected biomarker, we estimate just one.

**Fig. 8.**
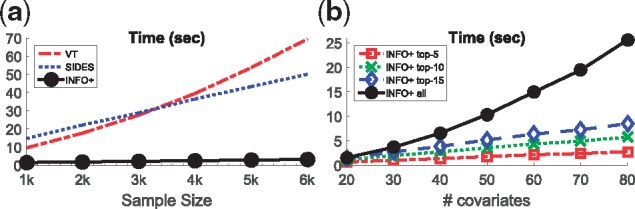
Comparing VT/SIDES/*INFO+ *in terms of their execution time. For *INFO+ *we have two versions, one that it returns a full ranking of *all* biomarkers, and one that it is just returns the top-*K* most important biomarkers. For all the experiments we simulated data from **M-1** with predictive strength *θ* = 1. (**a**) Execution time vs sample size. (**b**) Using *INFO*+ with various top-K. For (**a**) we fixed dimensionality *p* = 30 and we simulate various sample sizes, while for (**b**) we fixed sample size *n* = 2000 and we simulate various dimensionalities

Figure 8a shows that our optimized version of *INFO+ *outperforms all of the other methods for all sample sizes. Furthermore, by our step-wise forward selection we can improve the computational cost, by just asking to return the top-*K* biomarkers instead of the full ranking. This is very useful in practice, where we have high-dimensional data where only few variables contain meaningful information. Figure 8b shows the execution time for various values of top-*K* biomarkers, using our optimized version of *INFO+*. As we observe, we can save computational time by just returning the most important predictive biomarkers instead of ranking all of them. This result can be very useful in high dimensional trials.

**Remark 8:** Our optimized implementation of *INFO+ *is the most computationally efficient way to derive full rankings. Furthermore, by our forward step-wise procedure, *INFO+ *is suitable for exploring the ranking of the top-*K* most influential biomarkers, something very useful for high-dimensional trials.

### 3.2 Applications to real clinical trials

Now we will present two applications of our methods in real clinical trials, and introduce a new graphical representation that provides more insight into the prognostic and predictive strength of each biomarker.

#### 3.2.1 Lung cancer study: IPASS trial

It is of interest to explore how the suggested methods perform on a real clinical trial data, which has a known predictive biomarker. We explore the IPASS study (Mok *et al.*, 2009): a Phase III, multi-center, randomized, open-label, parallel-group study comparing *gefitinib* (Iressa, AstraZeneca) with carboplatin (Paraplatin, Bristol-Myers Squibb) plus paclitaxel (Taxol, Bristol-Myers Squibb) as a first-line treatment for clinically selected patients from East Asia, who had advanced non small-cell lung cancer (NSCLC). The primary end point was progression-free survival (PFS).

**Fig. 9.**
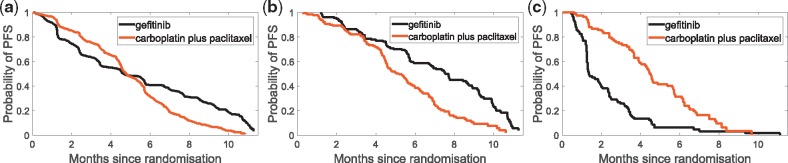
Kaplan–Meier curves for the probability of progression-free survival (PFS) for: (**a**) the overall population, where we see that the study met its primary objective and showed the superiority of gefitinib as compared with carboplatin-paclitaxel for PFS [Hazard Ratio (HR) = 0.74, 95% CI 0.65–0.85; *P* < 0.001]. Furthermore, EGFR mutation carries predictive information: (**b**) in the mutation positive subgroup patients treated with gefitinib had significantly longer PFS than the ones treated with carboplatin-paclitaxel (HR = 0.48, 95% CI 0.36–0.64; *P* < 0.001), while (**c**) in mutation negative subgroup, patients in carboplatin-paclitaxel group had longer PFS than the ones in gefitinib (HR = 2.82, 95% CI 2.03–3.94; *P* < 0.001). Note: as this is an unplanned analysis, all *P* values are nominal, and they have been used as descriptive measures of discrepancy and not as inferential tests of null hypotheses

It is known that gefitinib inhibits the epidermal growth factor receptor (EGFR), and is now indicated for the first-line treatment of patients with NSCLC whose tumours have specific EGFR mutations. Figure 9 presents the main finding of IPASS study (Mok *et al.*, 2009): the presence in the tumor of a mutation of the EGFR gene is strongly predictive for better outcome with gefitinib. We therefore expect EGFR mutation status to appear as a strongly predictive biomarker. A detailed description of the trial can be found in Section S8 of the Supplementary Material.

On the full 1217 subjects, all three of VT/SIDES/*INFO+*, identify EGFR mutation status as the most predictive biomarker—however, an interesting question is to explore how they perform with minor perturbations in the data. Table 2 presents, for each method, the top-3 biomarkers with the highest score, averaged over 500 bootstrap samples along with required computational time. As we see, *INFO+ *is an order of magnitude faster than the competing methods. Of the biomarkers seen in Table 2, it is reassuring that *INFO+ *suggests EGFR mutation status to be the most predictive; as discussed above gefitinib inhibits EGFR, which was noted to have a significant interaction with the treatment indicator in the original study (Mok *et al.*, 2009). Ethnicity is also related to the likelihood of EGFR mutation status; it is unsurprising that this has been pulled out by VT as a possible predictive biomarker, while our method, *INFO+*, manages to capture this interaction.

**Table 2.** Top-3 predictive biomarkers in IPASS for each competing method

Position	*INFO+*	VT	SIDES
1st	EGFR mut. status (*X*_2_)	Weight (*X*_5_)	WHO perf. status (*X*_1_)
2nd	Weight (*X*_5_)	EGFR mut. status (*X*_2_)	EGFR mut. status (*X*_2_)
3rd	Age (*X*_11_)	Ethnicity (*X*_7_)	Bilirubin (*X*_15_)
Time (s)	0.97	18.52	6.76

Now we will present a visualization tool, *PP*-graphs, that captures both the *prognostic* and *predictive* strength of biomarkers. It is our hope that this may provide useful information to healthcare professionals, in controlling false discoveries in clinical trials.

**Fig. 10.**
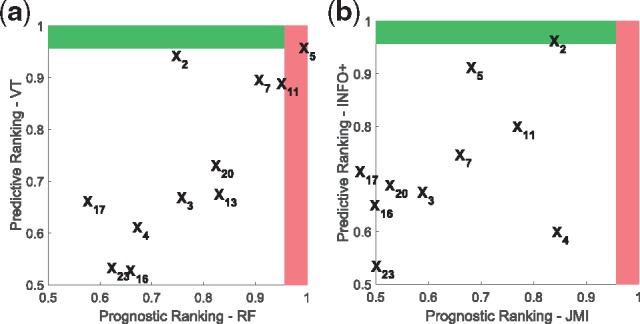
***PP*-graphs** for IPASS trial using two different approaches: (**a**) VT and RF: for this graph we used random forests to derive the prognostic score of each biomarker, and the counterfactual modelling of Virtual-Twins for the predictive score, (**b**) *INFO+ * and JMI: for this graph we used two information theoretic approaches that capture higher order interactions, JMI and *INFO+ *for the prognostic and predictive score respectively. Note that with our *INFO+ *the most predictive biomarker is *X*_2_ (EGFRMUT), which we know that carries predictive information

**Description of *PP*-graphs**: A *PP*-graph (Fig. 10) is a scatter plot, where each point represents a biomarker, while coordinates (*x*, *y*) capture its prognostic and predictive strength respectively. For example, one way to measure prognostic strength is to rank the biomarkers, and then use a normalized score that takes values in [0,1], where 1 is the score for the most-prognostic biomarker. To derive a prognostic ranking we can use the dataset ${\left\{x_{i},y_{i}\right\}}_{i=1}^{n}$ and any method that ranks biomarkers on their dependence with the output. For example, we can use any information theoretic method (Brown *et al.*, 2012), such as MIM/JMI, or we can use RF and rank the biomarkers on their variable importance score. On the other hand, to derive predictive rankings we can use the dataset ${\left\{x_{i},t_{i},y_{i}\right\}}_{i=1}^{n}$ and any method presented so far for deriving predictive rankings, such as *INFO*/*INFO+*/VT/SIDES/IT/MCR. Furthermore, we can capture the sample variations on the ranking scores by using a resampling methodology. For example, instead of estimating the scores only once from the whole dataset, we can average over the scores of a large number of bootstraps.

The red area (vertical shaded region) represents the top-*K* prognostic-biomarkers, while the green (horizontal shaded region) the top-*K* predictive. For example for the *PP*-graphs of Figure 10 we used k=1, which corresponds to the score cut-off value of (p−k)/p=(23−1)/23=0.96, where *p* = 23 is the total number of biomarkers in IPASS trial. The biomarkers being in the red (vertical shaded region) and green (horizontal shaded region) areas, are the ones that ranked, on average, in the first position of the prognostic and predictive ranking respectively. The intersection of these two areas—top right area—will contain the biomarkers that are both prognostic and predictive.

***PP*-graphs for RF biomarker discovery in IPASS**: Figure 10a shows the *PP*-graph of RF based methods. For the prognostic axis we used RF to rank the biomarkers, while for the predictive axis VT, which is a counterfactual modelling method based on RF. Furthermore, we plot the average predictive/prognostic normalized ranking scores over 500 bootstrap samples of IPASS dataset. Using VT, the top four predictive biomarkers (*y*-axis) are *X*_5_, *X*_2_, *X*_7_ and *X*_11_ (Supplementary Table S5 provides the names of the biomarkers). However, we could ask the question whether these biomarkers are also prognostic, and, by using RF, we observe that $X_{5},X_{11},X_{7}$ and *X*_13_ are the most prognostic biomarker (*x*-axis). Knowing the result from Section 3.1.4, that VT may be biased towards strongly prognostic biomarkers, we might now change our investigation: instead of pursuing *X*_5_ we should perhaps prioritize $X_{2}.$

***PP*-graphs for information theoretic biomarker discovery in IPASS**: Instead we can use the information theoretic methodology proposed in this paper, in which the predictive (*y*-axis) and prognostic (*x*-axis) rankings are derived from a *self-consistent* manner. For example, in Figure 10b, the ranking in the *y*-axis is derived by using *INFO+*, while the ranking in the *x*-axis by using JMI (Section 2.4). Both of these approaches capture higher order interactions, by using low dimensional approximations. This *PP*-graph shows that our suggested *INFO+ *approach correctly ranks as the most important predictive biomarker *X*_2_ (green area, horizontal shaded region).

**Fig. 11.**
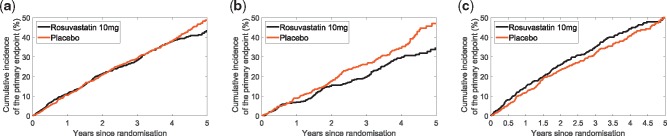
Kaplan–Meier curves for the cumulative incidence of the primer end point in the two study groups for: (**a**) the overall population, where we see that the study did not met its primary objective since treatment with rosuvastatin was not associated with a reduction in major adverse cardiac events (HR = 0.95, 95% CI 0.83–1.10; *P* = 0.516). In (**b**) we can see that Lymphocytes may carry a predictive information, since in the 994 patients with low percent lymphocytes (<65%) those who were treated with rosuvastatin had much longer MACE-free survival than the ones taking the placebo (HR = 0.78, 95% CI 0.61–0.99; *P* = 0.037). On the other hand, in (**c**) we see that for patients with high percent lymphocytes (>= 65%) there is no evidence of predictive information (HR = 1.08, 95% CI 0.90–1.29; *P* = 0.415). Note: as this is an unplanned analysis, all *P* values are nominal, and they have been used as descriptive measures of discrepancy and not as inferential tests of null hypotheses

#### 3.2.2 Cardiovascular disease study: Aurora trial

Another interesting scenario to explore is how our methods perform in a trial where there is no known predictive biomarker. We explore the AURORA study (Fellström *et al.*, 2009): a randomized, double-blind, placebo-controlled, multicenter trial in which 2776 patients with end-stage renal disease were randomly assigned 1:1 to double-blind treatment with rosuvastatin at a dose of 10 mg or placebo. The primary endpoint was the time to a major cardiovascular event (MACE) defined as a nonfatal myocardial infarction, nonfatal stroke, or death from cardiovascular causes. All myocardial infarctions, strokes and deaths were reviewed and adjudicated by a clinical end-point committee whose members were unaware of the randomized treatment assignments, in order to ensure consistency of the event diagnosis. A detailed description of the trial can be found in Section S9 of the Supplementary Material. For full details of the trial see (Fellström *et al.*, 2009).

Figure 11a presents Kaplan–Meier curves of the cumulative incidence of the primary end point (MACE) in the overall population, where we see that the study failed to meet its primary objective: treatment with rosuvastatin was not associated with a reduction in major adverse cardiac events (HR = 0.95, *P* =0.516). Furthermore, rosuvastatin had no benefit in any examined subgroup, more details can be found in (Fellström *et al.*, 2009). It is important to have a structured way to explore the data in such trials, in which any hypotheses arising out of the data may be handled in a controlled manner.

Table 3 presents, for each predictive biomarker discovery method VT/SIDES/*INFO+*, the top-3 biomarkers with the highest score, averaged over 500 bootstrap samples. None of the suggested variables have been previously identified as predictive, although Age has previously been identified as prognostic in a post hoc analysis (Schneider *et al.*, 2013).

**Table 3.** Top-3 predictive biomarkers in AURORA for each competing method

Position	*INFO+ *	VT	SIDES
1st	Lymphocytes (*X*_24_)	Age (*X*_1_)	Hematocrit (*X*_21_)
2nd	Apolipoprotein B (*X*_30_)	BMI (*X*_7_)	Haemoglobin (*X*_22_)
3rd	Leukocyte conc. (*X*_23_)	Pulse pres. (*X*_15_)	Leukocyte conc. (*X*_23_)

**Fig. 12.**
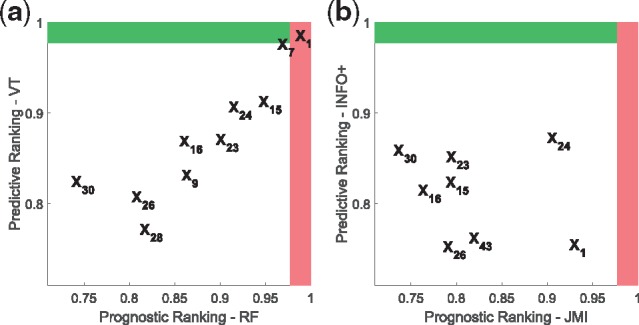
***PP*-graphs** for AURORA trial using two different approaches: (**a**) for this graph we used random forests to derive the prognostic score of each biomarker, and the counterfactual modelling of Virtual-Twins for the predictive score, (**b**) for this graph we used two information theoretic approaches that capture higher order interactions, JMI and *INFO+ *for the prognostic and predictive score respectively. Note that only VT ranks a biomarker (*X*_1_) in the predictive area

As in the IPASS trial, it is also informative to explore the prognostic strength of each biomarker. Figure 12 presents the *PP*-graphs for AURORA trial. As earlier, the red area (vertical shaded region) represents the top-*K* prognostic-biomarkers, while the green (horizontal shaded region) the top-*K* predictive. For the *PP*-graphs of Figure 12 we used again k=1, which corresponds to the score cut-off value of (p−k)/p=(44−1)/44=0.9773, where *p* = 44 is the total number of biomarkers in the trial. The biomarkers being in the red (vertical shaded region) and green (horizontal shaded region) areas, are the ones that ranked, on average, in the first position of the prognostic and predictive ranking respectively. Figure 12a shows that only VT ranks a biomarker in the predictive area. VT ranks *X*_1_ (Age) as the most predictive biomarker, but the same biomarker also carries the most prognostic information. Taking into account the previously observed bias of VT to prognostic biomarkers, we might conclude that age is a false positive. On the other hand, Figure 12b shows that our suggested method, *INFO+*, does not rank any biomarker close to the predictive region (green area, horizontal shaded region)—a result in agreement with the trial findings.

At this point it is useful to explore more the biomarker that *INFO+ *returned as the most predictive, the percent of lymphocytes (*X*_24_) in the blood. Interestingly, in the subgroup of 994 patients with low percentage (< 65%) (Fig. 11b) the ones receiving rosuvastatin they had longer MACE-free survival than the ones receiving placebo (HR = 0.78, *P* =0.037).

The *INFO+ *method has identified inflammatory status (lymphocytes & leukocytes) as predictive markers, which is a new and unvalidated hypothesis, which did not surface in the AURORA trial. For example, the subgroup of Figure 11b was 994 patients, a non-trivial subgroup size in a trial of this nature. The patient population is, though, known to be complicated, and further research is necessary to assess the plausibility of the suggested biomarkers. There are previously noted prognostic associations for cardiovascular events in the literature (Xiang *et al.*, 2018), but no investigation of predictive nature with Rosuvastatin. Such a future investigation seems plausible to yield interesting results, but we do not claim any association from this dataset/paper alone—as always, methods such as *INFO+*, are exploratory rather than confirmatory.

## 4 Conclusions

The primary contribution of this work is a formalism for data-driven ranking of predictive versus prognostic biomarkers. With an information theoretic approach, we can disentangle the prognostic versus predictive strength of a biomarker, naturally allowing for issues such as correlated biomarkers. We presented a novel procedure for predictive biomarker discovery, *INFO+*, which we evaluated over a wide gamut of synthetic data, increasing in difficulty. Our results demonstrate that *INFO+ *captures higher order interactions between biomarkers without the need to explicitly model the functional form of the predictive part. Furthermore, it disentangles better the predictive and prognostic information of each biomarker, and as a result, provides a better performance in terms of TPR/${\text{FNR}}_{\text{Prog}.}.$ The *INFO+ *approach also requires 1–3 orders of magnitude less in computation, compared to appropriate baselines, making it feasible to explore datasets larger than ever before. Furthermore, we introduced a new visual representation, the *PP*-graph, that captures both the prognostic and the predictive strength of a set of biomarkers. We expect that this tool will prove beneficial in visualizing and interpreting biomarker investigations for clinical trials. Finally, by formalizing the problem of predictive biomarker discovery in information theoretic terms, we can potentially extend this work to other challenging scenarios, such as misclassification bias (Sechidis *et al.*, 2017) or partially labelled data (Sechidis and Brown, 2018).

## Supplementary Material

Supplementary Data
