# Distinguishing prognostic and predictive biomarkers: an information theoretic approach

**DOI:** 10.1093/bioinformatics/bty515

**Published:** 2018-07-19

**Authors:** Konstantinos Sechidis, Konstantinos Papangelou, Paul D Metcalfe, David Svensson, James Weatherall, Gavin Brown

*Bioinformatics,* (2018) https://doi.org/10.1093/bioinformatics/bty357

The author wishes to apologize for a mistake in Figure 2 in the above manuscript. The figure appears correctly below:

**Fig. 2.**
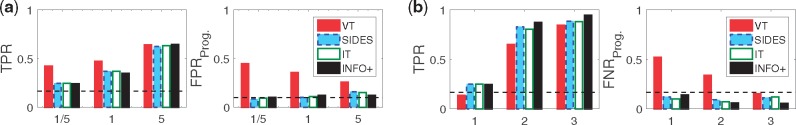
When biomarkers have both prognostic/predictive strength (M-1) VT achieves higher TPR, otherwise (M-2) the gains in TPR are vanishing. In terms of ${\text{FNR}}_{\text{Prog}.}$, VT always has very high error rate on selecting *solely* prognostic biomarkers as predictive, and it performs worse than random selection. This is the average TPR/${\text{FNR}}_{\text{Prog}.}$ over 200 simulated datasets for three different values of the predictive strength *θ*: 1/5 means a strongly prognostic signal, 1 means equal strength between prognostic and predictive signals, and 5 means a strongly predictive signal. The sample size is 2000, and the dimensionality *p* = 30 biomarkers. Dashed lines show the TPR/${\text{FNR}}_{\text{Prog}.}$ if we were ranking the biomarkers at random. (**a**) **M-1:** Biomarkers can be both prognostic and predictive. (**b**) **M-2:** Biomarkers are solely either prognostic or predictive

The paper has been corrected online.

