# Supplementary material for: Distinguishing prognostic and predictive biomarkers: an information theoretic approach
Source: Bioinformatics. 2018 May 2;34(19):3365–76. doi: 10.1093/bioinformatics/bty357 (PMC6157098; doi:10.1093/bioinformatics/bty357)
Supplement: Supplementary Data [file bty357_supplementary_material.pdf]

OXFORD

Supplementary material

# Distinguishing prognostic and predictive biomarkers: An information theoretic approach

**Konstantinos Sechidis [1,*], Konstantinos Papangelou [1], Paul D. Metcalfe [2], David Svensson [2], James Weatherall [2] and Gavin Brown [1]**

[1] School of Computer Science, University of Manchester, Manchester, M13 9PL, UK and
[2] Advanced Analytics Centre, Global Medicines Development, AstraZeneca, Cambridge, SG8 6EE, UK.

*To whom correspondence should be addressed.

## 1 Background on predictive biomarker discovery

The problem of finding *predictive biomarkers* is closely related to the problem of data-driven subgroup identification, for which an excellent review is recently provided by Lipkovich *et al.* (2017) — we present relevant literature following their suggested taxonomy.

- **Global outcome modelling methods**, *which model the outcome function* $f(\boldsymbol{X}, T)$.
  The simplest way to model this function is by using generalised linear regression models (Freidlin and Simon, 2005; Tian *et al.*, 2014). Different types of extensions have been suggested in the literature, such as FindIt (Imai and Ratkovic, 2013), which uses a support vector machine (SVM) classifier with differing penalisation for the predictive vs the prognostic covariates. Another category of methods utilises the potential outcome (or counterfactual) modelling (Rubin, 1974). A popular method in this class is Virtual Twins (VT) (Foster *et al.*, 2011), which follows a three-step strategy. Firstly, it non-parametrically estimates the outcome function $f(\boldsymbol{X}, T)$ using random forests (RF) (Breiman, 2001). Then, it estimates the treatment effect (i.e. treatment difference) for each patient $i$: $\widehat{z}(\boldsymbol{x}_i) = \widehat{f}(\boldsymbol{x}_i, 1) - \widehat{f}(\boldsymbol{x}_i, 0)$. Finally, it identifies interesting subgroups by building a regression tree, using $\boldsymbol{X}$ as an input, and the treatment difference $Z$ as target variable.
- **Global treatment effect modelling methods**, *which directly model the treatment effect* $z(\boldsymbol{X})$.
  One of the earliest subgroup identification method is Interaction Trees (IT) (Su *et al.*, 2008), an extension of the CART decision tree algorithm (Breiman *et al.*, 1984) that utilizes the treatment variable. The splitting criterion in IT is an interaction test between treatment and covariate, and as a result, IT splits the data in such a way that the heterogeneity with respect of the treatment contrast $z(\boldsymbol{X})$ is increased. Loh *et al.* (2015) proposed a different method, the Generalized Unbiased Interaction Detection and Estimation (GUIDE) tree, which overcomes one of the inherent biases of CART: favouring variables with a large number of categories. While all the above methods are non-parametric, the treatment effect can be also modelled with parametric methods. For example, Tian *et al.* (2014) present a method where they fit a multivariate regression to predict the outcome, by using as input the modified covariates $z(\boldsymbol{X})T/2$ (where the coding for the treatment $T$ variable is $\pm 1$), and *without* the need of modelling the main effects.
- **Local modelling methods**, *which directly searches for subgroups with improved treatment effect* $z(\boldsymbol{X})$.
  Kehl and Ulm (2006) presented a method to solve the problem of responder identification in censored data by extending the bump hunting procedure (Friedman and Fisher, 1999). Another popular algorithm is the Subgroup Identification based on Differential Search (SIDES) (Lipkovich *et al.*, 2011), which directly searches for specific areas in the covariate space with an enhanced treatment-by-biomarker interactions. SIDES is a recursive partitioning method inspired by bump hunting.

For the experiments in our paper, we selected representative methods from each category: Virtual Twins (global outcome modelling), Interaction Trees and Modified Covariate Regression (global treatment modelling), and SIDES (local modelling). The primary objective of all these is *subgroup* discovery. We remind the reader that our primary objective is distinguishing prognostic/predictive markers by ranking them according to their effect. Some of the methods above, such as SIDES and IT, do quantify predictive strength via variable importance scores, and these scores can be used to derive predictive biomarker rankings. Some other methods, such as VT/MCR, can be straightforwardly adapted to generate predictive biomarker rankings (more details in Section 5 os the Supplementary material).

These methods can also be discussed/characterised from the perspective of the statistical tools each is using: penalised linear regression methods (such as MCR), counterfactual modelling methods (such as VT) and recursive partitioning methods (such as SIDES/IT).

In our article we use the tool of *information theory* to introduce a new approach that explicitly disentangles the prognostic and predictive strength of each biomarker. By using our new formalization of the problem we suggest two novel methods, *INFO*/*INFO+*, for predictive biomarker ranking. The methods also fit neatly into the second category of Lipkovich *et al.* (2017), as a global treatment effect modelling method and, in summary, the following advantages hold:

© The Author 2015. Published by Oxford University Press. All rights reserved. For permissions, please e-mail: journals.permissions@oup.com

1. Unlike penalised linear regression methods, our methods do not assume any functional form for the interaction and the main effects. Main paper's Section 3.1.5 explores empirically this result, and shows that *INFO+* captures high-order biomarker interactions, without assuming any specific predictive model.
2. Unlike potential outcome modelling methods, our methods do not build a prediction model to derive the treatment effect. To derive this effect, the potential outcome methods build a prediction model that infers the outcome of each patient under opposite treatment assignment and as a result, they make further modelling assumptions (Gelman and Hill, 2006, Chapter 9). There are scenarios where this counterfactual modelling may fail. For example, in the first step of VT, the classifiers that we use to estimate $\widehat{f}(\boldsymbol{x}, t)$ may ignore completely the treatment variable, and just focus on the strongly prognostic biomarkers. Main paper's Section 3.1.4 explores empirically these scenarios, and shows that counterfactual modelling through VT is biased towards strongly prognostic biomarkers, while on the other hand, information theoretic modelling through *INFO+* achieves a better trade-off between true positive and false positive decisions.
3. Unlike recursive partitioning methods, our methods use *all* the available data in *every* step for estimating the predictive strength and for capturing possible interaction between the biomarkers. To do this in a sample efficient way, we derive low dimensional approaches in combination with shrinkage estimators that are suitable for 'small-sample size, high-dimensionality' scenarios (Hausser and Strimmer, 2009). Main paper's sections 3.1.6 and 3.1.7 explore empirically these scenarios, by comparing the performance of the different methods for different sample-sizes and different dimensionalities. Furthermore, Main paper's Section 3.1.8 explores the scenario of having subgroups of patients with an enhanced treatment effect.
4. Unlike all the previous methods that derive full rankings of all biomarkers, our approach exploits a step-wise procedure that can be used to derive partial rankings (i.e. top-$K$ predictive biomarkers). This brings huge improvement in terms of the computational cost. Main paper's Section 3.1.10 compares the different methods in terms of their computational complexity.

## 2 Background on information theory

In information theory, a fundamental concept is the *mutual information*[1] between two random variables $X$ and $Y$ (Shannon, 1948; Cover and Thomas, 2006; Steuer *et al.*, 2002):

$$I(X;Y) = \int_X \int_Y p(x,y) \log \frac{p(x,y)}{p(x)p(y)}\, dxdy.$$

Mutual information quantifies the amount of information two variables share. The choice of the base of the logarithm merely alters the scale: popular choices are 2, giving a value in *bits*, and $e$ (natural logarithm) giving a value in *nats*. The above is the generic phrasing, where $x, y$ could be continuous or discrete valued. In the discrete data scenario, the integrals become sums over the possible values: for example the set $\{0,1\}$ if $Y$ is a binary outcome variable. In a medical scenario the random variable $X$ might represent some patient factors, and $Y$ a binary health outcome. The mutual information quantifies the dependence between random variables – larger if they are dependent, or zero if statistically independent. In a practical situation, we imagine that our dataset $\{\boldsymbol{x}_i, y_i\}_{i=1}^n$ is a sample from the joint distribution $p(x,y)$ of these random variables, and we estimate the relevant probabilities. When $x, y$ are discrete valued, it is common to use maximum likelihood estimates (simple frequency counts), and the estimator is in general denoted:

$$\hat{I}(X;Y) = \sum_{x\in\mathcal{X}, y\in\mathcal{Y}} \hat{p}(x,y) \log \frac{\hat{p}(x,y)}{\hat{p}(x)\hat{p}(y)}$$

A second important concept for this paper is the *conditional mutual information*, which is:

$$I(X;Y|Z) = \sum_{x\in\mathcal{X}, y\in\mathcal{Y}, z\in\mathcal{Z}} p(x,y,z) \log \frac{p(x,y|z)}{p(x|z)p(y|z)} \quad (1)$$

This quantifies the dependence between $X$ and $Y$, once we already know the value of a third variable $Z$. It can also more formally be stated as the expectation of $I(X;Y|Z=z)$ with respect to the distribution of $Z$. Again, the conditional information is zero when $X, Y$ are conditionally independent given $Z$, increasing with stronger dependence. In practice, when we have categorical data, we can estimate the conditional mutual information by using maximum likelihood estimates for the probabilities. This approach is known to have high error, especially in small-sample sizes (Steuer *et al.*, 2002). In Section 4 we will present a *shrinkage* method (Efron, 2012; Hausser and Strimmer, 2009) to estimate conditional mutual information, which achieves smaller error than the maximum likelihood approach, especially in small-sample scenarios.

Information theory provides an algebra with a well-defined set of semantics. For example if we have two features $x_1$, $x_2$, and denote their joint random variable as the concatenation $X_1X_2$, the following identity holds:

$$I(X_1X_2;Y) = I(X_1;Y) + I(X_2;Y|X_1) \quad (2)$$

This is known as the *chain rule* (Cover and Thomas, 2006), showing that the information about the outcome $Y$ contained in the joint $X_1X_2$ can be decomposed into the *individual* information in $X_1$, plus a conditional term accounting for its interaction with $X_2$.

## 3 Mutual information and log-linear models

In this section we will connect mutual information with log-linear models. The main outcome of this is to show that $I(T;Y|X)$ captures *only the predictive strength* and does not contain any prognostic information.

### 3.1 Log-linear models and independence in two-way tables

We will present that connection by using the notation of (Agresti, 2013, p. 339). Firstly, let us assume that we have two variables $X$ (biomarker) and $Y$ (target). For all probabilities, $\{\pi_{ij}\}$ the expected counts are: $\{\mu_{ij} = n\pi_{ij}\}$. For multinomial sampling, under statistical independence, the $\{\mu_{ij}\}$ have the structure:

$$\mu_{ij} = n\pi_{i+}\pi_{+j}$$

Since the independence formula is multiplicative, $\log \mu_{ij}$ has additive form:

$$M_0 : \log \mu_{ij} = \lambda + \lambda_i^Y + \lambda_j^X,$$

where the superscripts $X$ and $Y$ represent the variables and are not exponents. This is the *loglinear model of independence ($M_0$)*. The ML fitted values are $\{\mu_{ij} = \frac{n_{i+}n_{+j}}{n}\}$ the estimated expected frequencies for the $X^2$ test of independence.

[1] Note that the mutual information is symmetric, $I(X;Y) = I(Y;X)$.

Statistically dependent variables satisfy a more complex log-linear model:

$$M_1 : \log \mu_{ij} = \lambda + \lambda_i^Y + \lambda_j^X + \lambda_{ij}^{YX}$$

This is the *saturated model ($M_1$)*. The $\{\lambda_{ij}^{YX}\}$ are associated terms that reflect deviations from independence. The model $M_1$ is hierarchical, which means that the model includes all lower-order terms composed from variables contained in a higher order term. A reason for including lower-order terms is that, otherwise, the statistical significance and the interpretation of a higher order term depends on how variables are coded. This is undesirable and with hierarchical models the same results occur no matter how variables are coded.

The goodness-of-fit of the model of independence is measured by the *deviance*, which is the likelihood-ratio statistic for testing the null hypothesis that the model holds against the general alternative (i.e. the saturated model). In the log-linear model of independence the deviance can be written in terms of the mutual information as:

$$\text{Deviance} = G^2(M_0) - G^2(M_1) = 2NI(X;Y)$$

So the mutual information, that quantifies the dependence between $X$ and $Y$, it can be seen as a measure that quantifies how well the *model of independence* $M_0$, which is the model with the interaction terms, fits the data compared to the surrogate model. This is the exact definition of mutual information, the KL divergence between the joint $p(x,y)$ (surrogate model) and the product of the marginals $p(x)p(y)$ (independence model):

$$I(X;Y) = \mathbb{E}[\log \frac{p(x,y)}{p(x)p(y)}]$$

**Prognostic biomarker rankings by using $I(X;Y)$:** We can see that $I(X;Y)$ quantifies how close the two models $M_0$ and $M_1$ are. Since the only difference between these two models is the interaction term $\lambda_{ij}^{YX}$ – the "prognostic" term– the mutual information quantifies if there is some interaction between $X$ and $Y$. For that reason, we can derive prognostic biomarker rankings by ranking the biomarkers on the information that they share with the target.

## 3.2 Log-linear models and independence in three-way tables

Let us assume now that apart from biomarker $X$ and target $Y$ we also have a third variable, i.e. the treatment $T$. This is more complicated case, because there are several potential types of independence. In this section we will explore the conditional independence of $T$ and $Y$ given $X$, and we will explain why using $I(T;Y|X)$ to derive predictive biomarker rankings.

The saturated log-linear model for three-way tables is:

$$M_1' : \log \mu_{ijk} = \lambda + \lambda_i^Y + \lambda_j^X + \lambda_k^T + \lambda_{ij}^{YX} + \lambda_{kj}^{TX} + \lambda_{ik}^{TY} + \lambda_{ijk}^{TYX}$$

The model of conditional independence of $T \perp\!\!\!\perp Y|X$ can be derived by setting to zero the interaction co-efficents $\lambda_{ik}^{TY}$ and $\lambda_{ijk}^{TYX}$ (Agresti, 2013, Table 9.1)

$$M_0' : \log \mu_{ijk} = \lambda + \lambda_i^Y + \lambda_j^X + \lambda_k^T + \lambda_{ij}^{YX} + \lambda_{kj}^{TX}$$

The deviance of the log-linear model of conditional independence can be written in terms of the conditional mutual information as:

$$\text{Deviance} = G^2(M_0') - G^2(M_1') = 2NI(T;Y|X)$$

**Predictive biomarker rankings by using $I(T;Y|X)$:** From all the above it is shown that $I(T;Y|X)$ quantifies how close the two models $M_0'$ and $M_1'$ are. Since the only difference between these two models is the interaction terms $\lambda_{ik}^{TY}$ and $\lambda_{ijk}^{TYX}$ the conditional mutual information quantifies if there is some interaction between $T$ and $Y$. This information *does not* take into account any prognostic information, because the prognostic term $\lambda_{ij}^{YX}$ takes part in **both** models $M_0'$ and $M_1'$ and as a result it cancels out its effect. An interesting point to mention here is that this conditional mutual information quantifies *also* the effect of the term of the overall treatment effect $\lambda_{ik}^{TY}$ but since this term is independent of $X$ will not affect the ranking. It could be argued that the predictive strength should be normalised by subtracting the overall treatment effect, i.e. $I(T;Y|X) - I(T;Y)$. Since the normalising factor, $I(T;Y)$, does not depend on the biomarker $X$, both normalised and un-normalised versions will rank markers in exactly the same order.

Main paper's Section 3.1.3 shows experimentally that the conditional mutual information between the treatment and the outcome given a biomarker captures *only* the predictive strength of the biomarker.

# 4 Estimating Conditional Mutual Information

*INFO+* derives predictive biomarker rankings by estimating conditional mutual information quantities. Here we will describe the methods we implemented in our software for estimating mutual information for different data types (e.g. categorical, continuous, time-to-event outputs).

## 4.1 Categorical data

Because in clinical trials most of the times we have small-samples, it is desirable to use estimators that are tailored to these situations. For the purpose of this work, we will focus on categorical data, and now we will present a shrinkage estimator, which is suitable for 'small $n$, large $p$' scenarios (Hausser and Strimmer, 2009), and it outperforms the conventional maximum likelihood approach, especially when we have small-sample sizes. The conditional mutual information can be estimated as:

$$\hat{I}(T;Y|\boldsymbol{X}) = \sum_{t\in\mathcal{T},y\in\mathcal{Y},\boldsymbol{x}\in\boldsymbol{X}} \hat{p}(t,y,\boldsymbol{x}) \log \frac{\hat{p}(t,y,\boldsymbol{x})\hat{p}(\boldsymbol{x})}{\hat{p}(t,\boldsymbol{x})\hat{p}(y,\boldsymbol{x})} \quad (3)$$

To estimate the above expression, we need a way to estimate the joint probability $\hat{p}(t,y,\boldsymbol{x})$. The simplest approach is to use the maximum likelihood estimator (i. e. frequencies of observed counts), $\hat{p}^{\text{ML}}(t,y,\boldsymbol{x}) = \frac{n_{t,y,\boldsymbol{x}}}{n}$, where $n_{t,y,\boldsymbol{x}}$ is the shorthand for the counts of the event: $T = t, Y = y, \boldsymbol{X} = \boldsymbol{x}$, and $n$ is the sample size. By plugging in these probabilities in eq. (3), we derive the maximum likelihood estimator $\hat{I}^{\text{ML}}(T;Y|\boldsymbol{X})$.

The overall size of sample space is $d = |\mathcal{T}| \times |\mathcal{Y}| \times |\boldsymbol{X}|$, and it increases as we increase the dimensionality of $\boldsymbol{X}$. In scenarios where $n >> d$ the maximum likelihood estimator is known to be optimal (Hausser and Strimmer, 2009). But otherwise, a scenario that often can happen in clinical trials, it is challenging to estimate reliably the probabilities and as a result the conditional mutual information. This happens because of the small number of observed counts in each event, or even worse, events that do not have any example (zero-counts).

To overcome this limitation, Hausser and Strimmer (2009) derived a shrinkage estimator, similar to James and Stein (1961), for the multinomial probabilities. The main idea is to average over two different models: one high-dimensional, which is a low-bias/high-variance estimator, and one low dimensional, which a high-bias/low-variance. In our work we will follow the convention of choosing as high-dimensional

estimator the maximum likelihood $\hat{p}^{\text{ML}}(t, y, \boldsymbol{x}) = \frac{n_{t,y,\boldsymbol{x}}}{n}$, while as a low-dimensional estimator uniform probabilities $p^{\text{Uni}}(t, y, \boldsymbol{x}) = \frac{1}{d}$. The shrinkage estimator is then the convex combination:

$$\hat{p}^{\text{Shrink}}(t,y,\boldsymbol{x}) = \lambda p^{\text{Uni}}(t,y,\boldsymbol{x}) + (1-\lambda)\hat{p}^{\text{ML}}(t,y,\boldsymbol{x}), \forall t \in \mathcal{T}, y \in \mathcal{Y}, \boldsymbol{x} \in \boldsymbol{X}. \quad (4)$$

Schäfer and Strimmer (2005) derived a closed form expression for the optimal shrinkage intensity $\lambda^*$, optimal in terms of mean squared error, which can be estimated through the following expression (Hausser and Strimmer, 2009):

$$\hat{\lambda}^* = \frac{1 - \sum_{t \in \mathcal{T}, y \in \mathcal{Y}, \boldsymbol{x} \in \boldsymbol{X}} \left(\hat{p}^{\text{ML}}(t, y, \boldsymbol{x})\right)^2}{(n-1) \sum_{t \in \mathcal{T}, y \in \mathcal{Y}, \boldsymbol{x} \in \boldsymbol{X}} \left(p^{\text{Uni}}(t, y, \boldsymbol{x}) - \hat{p}^{\text{ML}}(t, y, \boldsymbol{x})\right)^2}. \quad (5)$$

From eq. (4) and (5) we can estimate the shrinkage probabilities. It can be shown that there is a one to one correspondence between the shrinkage and the Bayes estimates, which means that shrinkage is an *empirical Bayes estimator* (Hausser and Strimmer, 2009). Hausser and Strimmer (2009) used the shrinkage probabilities for estimating entropy in order to learn gene association networks, while Scutari and Brogini (2012) used them to derive a test of independence, in order to learn the structure of Bayesian networks. In our work by plugging the shrinkage probabilities in eq. (3) we derive a shrinkage estimator for the conditional mutual information $\hat{I}^{\text{Shrink}}(T; Y|\boldsymbol{X})$, which we will use to derive predictive biomarker rankings through *INFO* and *INFO+* (Main paper's Algorithm 1, Line 4).

Figure 1 compares the performance of the maximum likelihood and shrinkage estimator in terms of Mean Squared Error (MSE). To perform these experiments, we generated synthetic data with known conditional mutual information population values $I(T; Y|\boldsymbol{X})$. We used three different levels: 0 nats (or in other words $T$ and $Y$ conditionally independent given $\boldsymbol{X}$), 0.01 nats, and 0.10 nats. Furthermore, we assumed that the treatment and the outcome variables are binary (i.e. $|\mathcal{T}| = |\mathcal{Y}| = 2$), while $|\boldsymbol{X}| = 25$. We can see $\boldsymbol{X}$ as the joint variable of two features that can take five distinct values, which is for example the conditional mutual information we need to estimate in *INFO+*, Main paper's Algorithm 1 Line 4. As we observe, the shrinkage estimator performs better (i.e. lower MSE) and converges much faster than the maximum likelihood estimator.

## 4.2 Continuous covariates

While in the experiments of the main paper we focused on categorical datasets, *INFO+* can be used with different types of data using different types of estimators. Now we will provide a short review of some popular Machine Learning approaches for estimating mutual information.

When we have continuous variables (covariates and/or output) one straightforward way for estimating mutual information is by making some *parametric* assumption. For example, Brillinger (2004) derives an estimator under the popular Gaussian modelling. A *non-parametric* way to estimate mutual information is by partitioning the continuous space into discrete bins, a method that it is also known as binning (Steuer *et al.*, 2002). This can be done by using some pre-specified fixed number of bins, or with adaptive partitioning methods, such as the Fraser – Swinney algorithm (Fraser and Swinney, 1986). A popular adaptive partitioning method is the Maximal Information Coefficient (MIC) (Reshef *et al.*, 2011), which is a measure of divergence that uses a normalised version of the mutual information, and selects the number of bins by picking the one that maximizes the mutual information. There are other types of non-parametric estimators of the mutual information, such as kernel based approaches (Gretton *et al.*, 2005) or nearest-neighbor estimator (Pál *et al.*, 2010).

In our software we implemented an estimator for the conditional mutual information, which is based on non-parametric density estimation procedure. The core idea is to transform the continuous variables to categorical using a method for histogram generation, such as the Scott or the Freedman - Diaconis rule (Keen, 2010, Chapter 6), and then to use the shrinkage estimator presented earlier.

## 4.3 Survival outcome

Another interesting scenario is having survival data (also known as time-to-event data, or censored data). Beirlant *et al.* (1997) present a histogram-based method for estimating entropy from censored data. Extending their idea, we can derive a histogram-based method to estimate mutual information in censored data. Let $s = 1, ..., S$ be the distinct times of observed events in either group. Then, for each timestep we have the outcome variable $Y(s)$. For each patient, the value of this random variable is 0 if there is no progression information at this time point for this patient, or the value of the outcome value, if we have the progression information. Finally, we estimate the conditional mutual information, by averaging $\hat{I}(T; Y(s)|X)$ across each time step $s$.

## 4.4 Normalised Conditional Mutual Information

When we have different types of variables (continuous, categorical variables with different levels) it is challenging to compare directly the values of the conditional mutual information. To overcome this issue, we should use normalised versions of the conditional mutual information, which take into account the diverse characteristics of each covariate (Vinh *et al.*, 2010). The literature on deriving normalised information theoretic measures is vast (Kvålseth, 2017). In our implementation, for each feature $X$, we normalised the conditional mutual information $\hat{I}(T; Y|X)$ by dividing it with the conditional entropy $\sqrt{\hat{H}(T|X)\hat{H}(Y|X)}$.

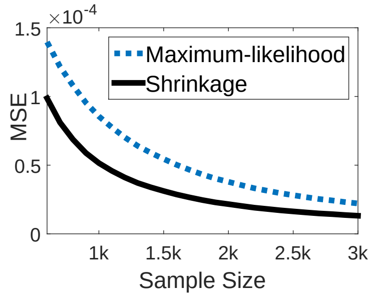


(a) $I(T; Y|\boldsymbol{X}) = 0$ nats

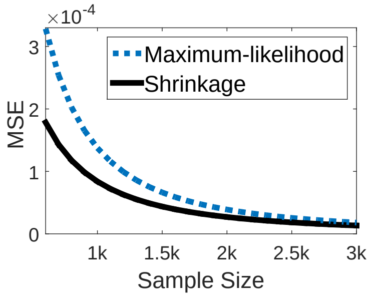


(b) $I(T; Y|\boldsymbol{X}) = 0.01$ nats

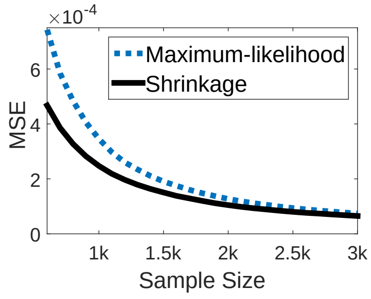


(c) $I(T; Y|\boldsymbol{X}) = 0.10$ nats

**Fig. 1.** Comparison in terms of MSE for the two estimators: maximum likelihood $\hat{I}^{\text{ML}}(T; Y|\boldsymbol{X})$ and shrinkage $\hat{I}^{\text{Shrink}}(T; Y|\boldsymbol{X})$. We plot the average over $10k$ iterations.

## 5 Competing methods

In this section we will provide all necessary implementation details.

- **MCR:** One way to rank the biomarkers on their predictive strength is by fitting a generalised linear regression model and explore the importance of the interaction terms. Recently, Tian *et al.* (2014) introduced the Modified Covariates Regression (MCR) method, where they fit the model with modified covariates $z(\boldsymbol{x})T/2$, *without* the need of modelling the main effects. The coding of the treatment $T$ variable is $\pm1$, and in the initial publication, the authors used $L_1$ regularization (Tibshirani, 1996) to perform variable selection. In our case we are interested in deriving rankings, for that reason it is more suitable to use $L_2$ regularization (i.e. a less sparse solution comparing to $L_1$ regularization) (Hoerl and Kennard, 1970). Furthermore, following Tian *et al.* (2014), to mimic the realistic situation where we do not have any knowledge about the true functional forms for interaction effects, we will assume a linear model $z(\boldsymbol{x}) = \boldsymbol{x}$. Lastly, to rank the strength of the different interaction terms we can use any generic methods for deriving variable importance from generalised linear regression models (Kuhn and Johnson, 2013). In our experiments to fit the penalised logistic regression models we used the R function *glmnet* (R package *glmnet* (Friedman *et al.*, 2010)), and we optimised the regularisation parameter using 10-fold cross validation and the one-standard-error rule – picking up the most parsimonious model within one standard error of the minimum (Hastie *et al.*, 2009). Then, we used the R function *varImp* (R package *caret* (Kuhn, 2008)) to derive a variable importance score for each biomarker, and using these scores we produced the predictive biomarker ranking.
- **VT:** Virtual Twins (Foster *et al.*, 2011) is a method that builds upon the counterfactual modelling idea of deriving the treatment difference for each example $i$ in the dataset: $z_i = \widehat{f}(\boldsymbol{x}_i, 1) - \widehat{f}(\boldsymbol{x}_i, 0)$. To derive this variable we used R package *aVirtualTwins* (Vieille, 2016) and train two Random Forest (RF) models in the two treatment groups. Following Foster *et al.* (2011), we fit the two models using all default parameters of the R function *randomForest*, except for the number of trees per forest which we set at 1000, and we also included the covariate-treatment interactions in the model. Finally, in the initial publication, the authors used the dataset $\{\boldsymbol{x}_i, z_i\}_{i=1}^n$ for subgroup identification by building a regression tree. In our work, since we are interested on deriving predictive rankings, we used this dataset to derive a variable importance score for each biomarker. A powerful method to derive variable importance scores (Hastie *et al.*, 2009) is RF, so we used again the R function *randomForest* to derive the scores. We produced the predictive biomarker ranking by using these scores.
- **SIDES:** As we presented in Section 1, a popular recursive partitioning method for subgroup discovery is SIDES (Lipkovich *et al.*, 2011). SIDEScreen (Lipkovich and Dmitrienko, 2014b) is an extension of SIDES, which is more suitable for high dimensional trials, and it can be used to derive a variable importance measure that captures the predictive power of each biomarker (Lipkovich and Dmitrienko, 2014a). Using these variable importance scores, which capture the relative predictive power of biomarkers, we can rank the biomarkers according to their predictive strength. In our experiments we used the R package *RSIDES*[2] to implement SIDEScreen, with the following parameters inspired by (Lipkovich *et al.*, 2017): splitting criterion differential, maximum number of promising child subgroups retained for each parent group (width) $M = 5$, maximum number of levels (depth) $L = 2$, smallest sample size in the any of the two child subgroups formed by a split 30, while we used the default values for the remaining parameters. Furthermore, since we are interested on the variable importance ranking we turned off the complexity control (Lipkovich and Dmitrienko, 2014a).
- **IT:** Another recursive partitioning technique is Interaction Trees (IT) (Su *et al.*, 2009). In our experiments[3] we used the following parameters to generate IT: 20 the minimum number of observations for claiming a terminal node, 5 the minimum number of observations in each treatment arm within either of the two child groups when splitting a parent node, and 10 the maximum tree depth. To derive variable importance scores we generated random forests of IT (Su *et al.*, 2008, Section 2.5) with 500 trees, and using these scores we derived the predictive biomarker ranking.
- ***INFO+/INFO*:** For both our methods, *INFO* and *INFO+*, a minor number of parameters is needed. The only necessary parameters are the ones involved in the estimation of conditional mutual information. In the experiments of the main paper we focus on categorical datasets, so we can use our shrinkage estimator presented in Section 4, which is parameter-free. Thus, to use *INFO* and *INFO+* in this scenario we do not need to set-up *any* parameter. The software that implements our proposed methods will be publicly available as R package.

## 6 Description of simulation models

Table 1 presents three 'easy' models. In M-1 the $X$s are generated as independent variables $X_j \sim N(0, 1),\ j = 1 \dots p$, and the model has a simple linear form with five prognostic $\boldsymbol{X}_{\text{Prog.}} = \{X_1, X_2, X_3, X_4, X_5\}$ and five predictive variables $\boldsymbol{X}_{\text{Pred.}} = \{X_4, X_5, X_6, X_7, X_8\}$, while the rest $\{X_9, \dots, X_p\}$ are irrelevant (i.e. noisy variables). Note that $\boldsymbol{X}_{\text{Prog.}} \cap \boldsymbol{X}_{\text{Pred.}} = \{X_4, X_5\}$, which means that these two biomarkers carry both predictive and prognostic information. This is considered 'easy' as a method could identify a predictive marker by capitalising on its prognostic nature. M-2 is considered slightly more challenging for deriving predictive rankings, since we have no overlap between predictive and prognostic markers. M-3 is yet more challenging, as we have *correlated* covariates. To simulate challenging scenarios we generate correlations both within and between covariates in the predictive, prognostic and irrelevant sets. In practice we generate the odd-numbered covariates { $X_1, X_3, X_5$... } to have an internal correlation of $\rho = 0.7$, and even-numbered, { $X_2, X_4, X_5$... } , to have the same internal correlation – i.e. given any two odd valued indices $i, j \in \{1 \dots p\}$ , the correlation between $X_i, X_j$ is $\rho = 0.7$, and the same holds for two even valued indices.

Table 2 presents two models of 'medium' difficulty. We still have five prognostic and five predictive variables, but this time we have non-linear interactions. In M-4 we have second order, while in M-5 we even have fourth order interaction terms. In both models the covariates are correlated in the same way as in M-3. Finally, Table 3 presents some 'hard' problems. M-6 has a predictive subgroup with an enhanced treatment effect, which is defined by thresholding two predictive biomarkers, $X_5$ and $X_6$. Threshold values are chosen such as $\sim 50\%$ of the data belong to the predictive subgroup. M-7 has a predictive subgroup with an enhanced treatment effect, but this time the subgroup is defined by *three* predictive biomarkers $X_6, X_7$ and $X_8$. Also, the threshold values are chosen such as $\sim 25\%$ of the data belong to the predictive subgroup.

Table 4 presents another interesting scenario, where we have a successful trial, since there is a significant effect of the treatment alone. In this case it exists an overall treatment effect in the population. We simulated two different models. **M-8** is an extension of the medium difficulty model **M-4** but this time it exists a significant effect if the treatment alone. **M-9**

[2] The code can be found on the Biopharmaceutical Network web site at:http://biopharmnet.com/subgroup-analysis/.

[3] Code available on the Biopharmaceutical Network web site.

Table 1. ‘Easy’ models: outputs $Y$ are generated by $\text{logit}[P(Y=1|T,\boldsymbol{X})] = f(\boldsymbol{X},T)$.

| ‘Easy’ models | $X$s correlated |
|---|---|
| **M-1**: $f(\boldsymbol{X},T) = \sum_{i=1}^{5} X_i + \theta T \sum_{j=4}^{8} X_j$ | ✗ |
| **M-2**: $f(\boldsymbol{X},T) = \sum_{i=1}^{5} X_i + \theta T \sum_{j=6}^{10} X_j$ | ✗ |
| **M-3**: $f(\boldsymbol{X},T) = \sum_{i=1}^{5} X_i + \theta T \sum_{j=6}^{10} X_j$ | ✓ |

Table 2. ‘Medium’ models: outputs are generated by $\text{logit}[P(Y=1|T,\boldsymbol{X})] = f(\boldsymbol{X},T)$, with correlated covariates.

| ‘Medium’ models |
|---|
| **M-4**: $f(\boldsymbol{X},T) = X_1 + X_2X_3 + X_4X_5 + \theta T(X_6 + X_7X_8 + X_9X_{10})$ |
| **M-5**: $f(\boldsymbol{X},T) = X_1\left(X_2X_3 - X_4^2\right) + \theta T X_5 \left(X_6X_7 - X_8^2\right)$ |

Table 3. ‘Hard’ models: outputs are generated by $\text{logit}[P(Y=1|T,\boldsymbol{X})] = f(\boldsymbol{X},T)$, with correlated covariates.

| ‘Hard’ models |
|---|
| **M-6**: $f(\boldsymbol{X},T) = X_1 + \mathbb{I}(X_2 > -0.545 \cap X_3 < 0.545) + \theta T(X_4 + \mathbb{I}(X_5 > -0.545 \cap X_6 < 0.545))$ |
| **M-7**: $f(\boldsymbol{X},T) = X_1 + \mathbb{I}(X_2 > -0.545 \cap X_3 < 0.545 \cap X_4 > 0) + \theta T(X_5 + \mathbb{I}(X_6 > -0.545 \cap X_7 < 0.545 \cap X_8 > 0))$ |

Table 4. Successful trial models, where it exists an overall treatment effect in the population. Outputs are generated by $\text{logit}[P(Y=1|T,\boldsymbol{X})] = f(\boldsymbol{X},T)$, with correlated covariates.

| Successful trials |
|---|
| **M-8**: $f(\boldsymbol{X},T) = 1 + X_1 + X_2X_3 + X_4X_5 + \theta T + \theta T(X_6 + X_7X_8 + X_9X_{10})$ |
| **M-9**: $f(\boldsymbol{X},T) = \alpha + X_1 + X_2X_3 + X_4X_5 + \theta\beta T + \theta T(X_6 + X_7X_8 + X_9X_{10}),\ \ \alpha,\beta \sim \mathcal{N}(0, 0.25)$ |

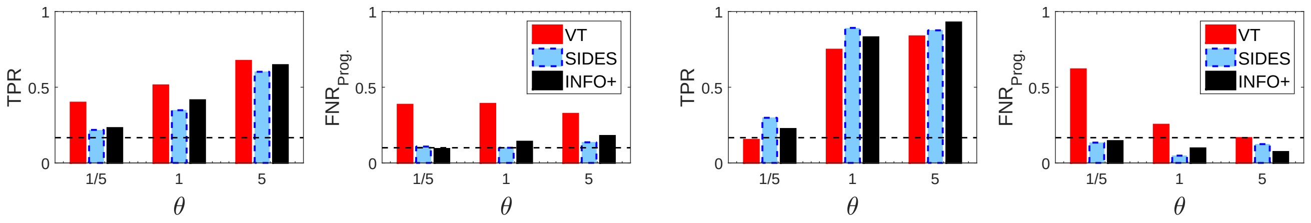


(a) **M-1**: Biomarkers can be *both* prognostic and predictive.

(b) **M-2**: Biomarkers are solely *either* prognostic or predictive.

**Fig. 2.** Same as Figure 2 of the main paper, but this time we are using continuous covariates.

is a different extension, inspired by (Foster *et al.*, 2011), where we have subject-specific effects generated by a normal distribution.

## 7 Experiments with continuous covariates

In this section we will repeat the experiments of Sections 3.1.4-3.1.8 of the main paper using continuous covariates. The implementations of SIDES and VT can handle continuous covariates, while for *INFO+*, we estimated the conditional mutual information by using the non-parametric histogram-based estimator presented in Section 4, using the Scott’s rule for non-parametric density estimation. Figures 2-6 are reproducing the results of Figures 2-7 of the main paper, but this time using continuous covariates instead of categorical.

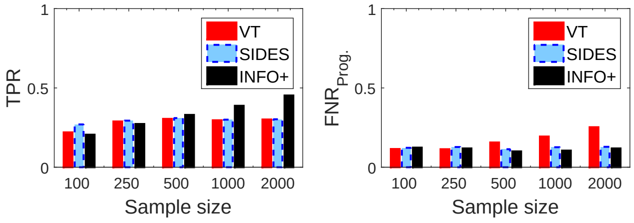


**Fig. 3.** Same as Figure 4 of the main paper, but this time we are using continuous covariates.

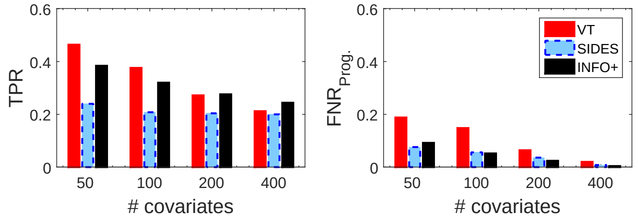


**Fig. 4.** Same as Figure 5 of the main paper, but this time we are using continuous covariates.

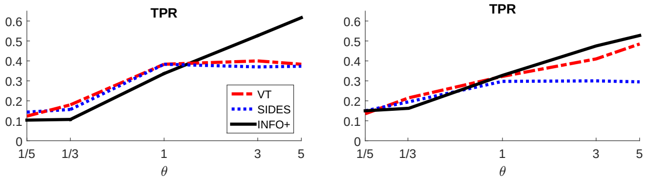


(a) **M-6**

(b) **M-7**

**Fig. 5.** Same as Figure 6 of the main paper, but this time we are using continuous covariates.

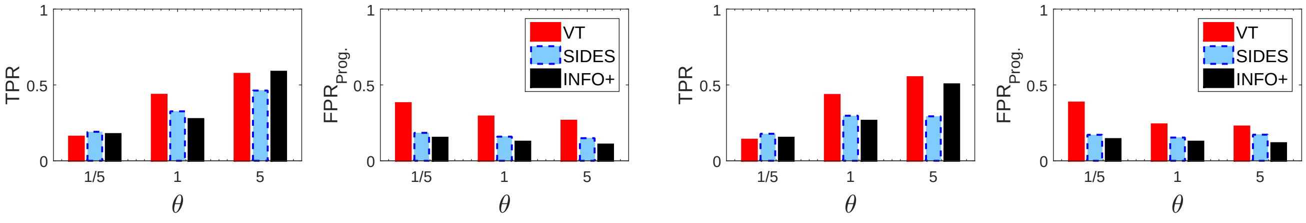


(a) **M-8**: Common treatment effect (b) **M-9**: Stochastic subject-specific treatment effect

**Fig. 6.** Same as Figure 7 of the main paper, but this time we are using continuous covariates.

## 8 Description of IPASS trial

IPASS study (Mok *et al.*, 2009; Fukuoka *et al.*, 2011) was a Phase III, multicenter, randomized, open-label, parallel-group study comparing gefitinib (Iressa, AstraZeneca) with carboplatin (Paraplatin, Bristol-Myers Squibb) plus paclitaxel (Taxol, Bristol-Myers Squibb) as first-line treatment in clinically selected patients in East Asia who had advanced non small-cell lung cancer (NSCLC). 1217 patients were randomized 1:1 between the treatment arms, and the primary end point was progression-free survival; for full details of the trial see (Mok *et al.*, 2009). It is known that gefitinib inhibits the epidermal growth factor receptor (EGFR), and is now indicated for the first-line treatment of patients with NSCLC whose tumours have specific EGFR mutations[4]. We therefore expect baseline EGFR mutation status to appear as a strongly predictive biomarker.

The covariates used in the IPASS study are shown in Table 5; for convenience we used categorical covariates as defined in the study. Our clinical endpoint is progression-free survival, and statistically we model this as a Bernoulli endpoint, neglecting its time-to-event nature. We analysed the data at 78% maturity, when 950 subjects have had progression events. The following covariates have missing observations (as shown in parentheses): $X_5$ (0.4%), $X_{12}$ (0.2%), $X_{13}$ (0.7%), $X_{14}$ (0.7%), $X_{16}$ (2%), $X_{17}$ (0.3%), $X_{18}$ (1%), $X_{19}$ (1%), $X_{20}$ (0.3%), $X_{21}$ (0.3%), $X_{22}$ (0.3%), $X_{23}$ (0.3%). Following Lipkovich *et al.* (2017), for the patients with missing values in biomarker $X$, we create an additional category, a procedure known as the *missing indicator method* (Allison, 2001).

Table 5. Covariates used in the IPASS clinical trial.

| Biomarker | Description | Values |
|---|---|---|
| $X_1$ | WHO perform. status | 0 or 1, 2 |
| $X_2$ | EGFR mutation status | Negative, Positive, Unknown |
| $X_3$ | EGFR FISH status | Negative, Positive, Unknown |
| $X_4$ | EGFR expression status | Negative, Positive, Unknown |
| $X_5$ | Weight | (0,50] , (50,60] , (60,70] , (70, 80] , (80, +∞) |
| $X_6$ | Race | Oriental, Other |
| $X_7$ | Ethnicity | Chinese, Japanese, Other Asian, Other not Asian |
| $X_8$ | Sex | Female, Male |
| $X_9$ | Smoking status | Ex-Smoker, Smoker |
| $X_{10}$ | Disease stage | Locally Advanced, Metastatic |
| $X_{11}$ | Age | (0, 44] , [45,64] , [65,74] , [75, +∞) |
| $X_{12}$ | Serum ALT | Low, Medium, High |
| $X_{13}$ | Serum ALP | Low, Medium, High |
| $X_{14}$ | Serum AST | Low, Medium, High |
| $X_{15}$ | Bilirubin | Low, Medium, High |
| $X_{16}$ | Calcium | Low, Medium, High |
| $X_{17}$ | Creatinine | Low, Medium, High |
| $X_{18}$ | Potassium | Low, Medium, High |
| $X_{19}$ | Sodium | Low, Medium, High |
| $X_{20}$ | Blood hemoglobin | Low, Medium, High |
| $X_{21}$ | Blood leukocytes | Low, Medium, High |
| $X_{22}$ | Blood neutrophils | Low, Medium, High |
| $X_{23}$ | Blood platelets | Low, Medium, High |

[4] www.accessdata.fda.gov/drugsatfda_docs/label/2015/206995s000lbl.pdf

## 9 Description of AURORA trial

AURORA was a randomized, double-blind, placebo-controlled, multicenter trial in which 2776 patients with end-stage renal disease were randomly assigned 1:1 to double-blind treatment with rosuvastatin at a dose of 10 mg or placebo. The primary endpoint was the time to a major cardiovascular event defined as a nonfatal myocardial infarction, nonfatal stroke, or death from cardiovascular causes. All myocardial infarctions, strokes, and deaths were reviewed and adjudicated by a clinical end-point committee whose members were unaware of the randomized treatment assignments, in order to ensure consistency of the event diagnosis. For full details of the trial see (Fellström *et al.*, 2009).

The 44 covariates used in the AURORA study are shown in Table 6. The covariates were mixed, i.e. both categorical and continuous. Following Lipkovich *et al.* (2017), for the the patients with missing values in a categorical covariate we created an additional category (i.e. missing indicator method), while for missing values in continuous covariates we replaced all missing data by the mean of that variable (i.e. mean imputation) (Allison, 2001). Furthermore, because some of the competing methods did not support mixed type (i.e. SIDES), to keep a fair comparison, the continuous variables were discretised in 5 levels following an equal-width strategy.

## References


Agresti, A. (2013). *Categorical Data Analysis*. John Wiley & Sons, 3 edition.

Allison, P. D. (2001). *Missing Data*. Sage University Papers Series on Quantitative Applications in the Social Sciences, 07-136.

Beirlant, J., Dudewicz, E. J., Györfi, L., and Van der Meulen, E. C. (1997). Nonparametric entropy estimation: An overview. *International Journal of Mathematical and Statistical Sciences*, **6**(1), 17–39.

Breiman, L. (2001). Random forests. *Machine learning*, **45**(1), 5–32.

Breiman, L., Friedman, J., Stone, C. J., and Olshen, R. A. (1984). *Classification and regression trees*. CRC press.

Brillinger, D. R. (2004). Some data analyses using mutual information. *Brazilian Journal of Probability and Statistics*, pages 163–182.

Cover, T. M. and Thomas, J. A. (2006). *Elements of information theory*. Wiley.

Efron, B. (2012). *Large-scale inference: empirical Bayes methods for estimation, testing, and prediction*. Cambridge University Press.

Fellström, B. C. *et al.* (2009). Rosuvastatin and cardiovascular events in patients undergoing hemodialysis. *New England Journal of Medicine*, **360**(14), 1395–1407.

Foster, J. C., Taylor, J. M., and Ruberg, S. J. (2011). Subgroup identification from randomized clinical trial data. *Statistics in Medicine*, **30**(24), 2867–2880.

Fraser, A. M. and Swinney, H. L. (1986). Independent coordinates for strange attractors from mutual information. *Physical review A*, **33**(2), 1134.

Freidlin, B. and Simon, R. (2005). Adaptive signature design: an adaptive clinical trial design for generating and prospectively testing a gene expression signature for sensitive patients. *Clinical Cancer Research*, **11**(21), 7872–7878.

Friedman, J., Hastie, T., and Tibshirani, R. (2010). Regularization paths for generalized linear models via coordinate descent. *Journal of statistical software*, **33**.

Friedman, J. H. and Fisher, N. I. (1999). Bump hunting in high-dimensional data. *Statistics and Computing*, **9**(2), 123–143.

Fukuoka, M. *et al.* (2011). Biomarker analyses and final overall survival results from a phase III, randomized, open-label, first-line study of gefitinib versus carboplatin/paclitaxel in clinically selected patients with advanced non–small-cell lung cancer in Asia (IPASS). *Journal of Clinical Oncology*, **29**(21), 2866–2874.

Table 6. Baseline covariates used in the AURORA clinical trial.

| Biomarker | Description | Biomarker | Description |
|---|---|---|---|
| $X_1$ | Age | $X_{23}$ | Blood leukocyte particle concentrations ($10^9$/L) |
| $X_2$ | Race or ethnic group | $X_{24}$ | Blood lymphocytes (%) |
| $X_3$ | Sex | $X_{25}$ | Blood monocytes (%) |
| $X_4$ | Current smoker | $X_{26}$ | Blood neutrophils (%) |
| $X_5$ | ACE use | $X_{27}$ | Serum alanine aminotransferase (IU/L) |
| $X_6$ | Beta-blocker use | $X_{28}$ | Serum albumin (g/L) |
| $X_7$ | Body mass index | $X_{29}$ | Serum apolipoprotein A-1 (g/L) |
| $X_8$ | CHD history | $X_{30}$ | Serum apolipoprotein B (g/L) |
| $X_9$ | DBP | $X_{31}$ | Serum apolipoprotein B/ apolipoprotein A-1 [ratio] |
| $X_{10}$ | Diabetic status | $X_{32}$ | Serum aspartate aminotransferase (IU/L) |
| $X_{11}$ | Dialysis treatment | $X_{33}$ | Serum calcium(mmol/L) |
| $X_{12}$ | Duration of dialysis | $X_{34}$ | Serum cholesterol (mmol/L) |
| $X_{13}$ | Years on heamodialysis trt. | $X_{35}$ | Serum cholesterol/ HDL cholesterol [ratio] |
| $X_{14}$ | Calculated Kt/V | $X_{36}$ | Serum creatine kinase (IU/L) |
| $X_{15}$ | Baseline pulse pressure | $X_{37}$ | Serum glucose, fasting (mmol/L) |
| $X_{16}$ | Baseline SBP | $X_{38}$ | Serum high-density lipoprotein (HDL) cholesterol (mmol/L) |
| $X_{17}$ | Baseline sevelamer use | $X_{39}$ | Serum LDL/HDL [ratio] |
| $X_{18}$ | Blood basophils (%) | $X_{40}$ | Serum low-density lipoprotein (LDL) cholesterol (mmol/L) |
| $X_{19}$ | Blood eosinophils (%) | $X_{41}$ | Serum non-HDL cholesterol (mmol/L) |
| $X_{20}$ | Blood erythrocyte particle concentration ($10^{12}$/L) | $X_{42}$ | Serum oxidised LDL (U/L) |
| $X_{21}$ | Blood hematocrit [ratio] | $X_{43}$ | Serum phosphate (mmol/L) |
| $X_{22}$ | Blood haemoglobin (g/L) | $X_{44}$ | Serum triglycerides (mmol/L) |


Gelman, A. and Hill, J. (2006). *Data analysis using regression and multilevel/hierarchical models*. Cambridge university press.

Gretton, A., Herbrich, R., Smola, A., Bousquet, O., and Schölkopf, B. (2005). Kernel methods for measuring independence. *Journal of Machine Learning Research*, **6**(Dec), 2075–2129.

Hastie, T., Tibshirani, R., and Friedman, J. (2009). *The elements of statistical learning: data mining, inference and prediction*. Springer, 2 edition.

Hausser, J. and Strimmer, K. (2009). Entropy inference and the james-stein estimator, with application to nonlinear gene association networks. *Journal of Machine Learning Research*, **10**(Jul), 1469–1484.

Hoerl, A. E. and Kennard, R. W. (1970). Ridge regression: Biased estimation for nonorthogonal problems. *Technometrics*, **12**(1), 55–67.

Imai, K. and Ratkovic, M. (2013). Estimating treatment effect heterogeneity in randomized program evaluation. *The Annals of Applied Statistics*, **7**(1), 443–470.

James, W. and Stein, C. (1961). Estimation with Quadratic Loss. In *Proceedings of the Fourth Berkeley Symposium on Mathematical Statistics and Probability, Volume 1: Contributions to the Theory of Statistics*, pages 361–379. University of California Press.

Keen, K. J. (2010). *Graphics for Statistics and Data Analysis with R*. Chapman & Hall/CRC, 1st edition.

Kehl, V. and Ulm, K. (2006). Responder identification in clinical trials with censored data. *Computational Statistics & Data Analysis*, **50**(5), 1338–1355.

Kuhn, M. (2008). Building Predictive Models in R Using the caret Package. *Journal of Statistical Software*, **28**.

Kuhn, M. and Johnson, K. (2013). *Applied predictive modeling*. Springer.

Kvålseth, T. O. (2017). On normalized mutual information: Measure derivations and properties. *Entropy*, **19**(11).

Lipkovich, I. and Dmitrienko, A. (2014a). Biomarker identification in clinical trials. In C. Carini, S. M. Menon, and M. Chang, editors, *Clinical and Statistical Considerations in Personalized Medicine*, chapter 8, pages 211–264. CRC Press.

Lipkovich, I. and Dmitrienko, A. (2014b). Strategies for identifying predictive biomarkers and subgroups with enhanced treatment effect in clinical trials using SIDES. *Journal of Biopharmaceutical Statistics*, **24**(1), 130–153.

Lipkovich, I., Dmitrienko, A., Denne, J., and Enas, G. (2011). Subgroup identification based on differential effect search - A recursive partitioning method for establishing response to treatment in patient subpopulations. *Statistics in Medicine*, **30**(21), 2601–2621.

Lipkovich, I., Dmitrienko, A., and D'Agostino Sr., R. B. (2017). Tutorial in biostatistics: data-driven subgroup identification and analysis in clinical trials. *Statistics in Medicine*, **36**(1), 136–196.

Loh, W.-Y., He, X., and Man, M. (2015). A regression tree approach to identifying subgroups with differential treatment effects. *Statistics in medicine*, **34**(11), 1818–1833.

Mok, T. S. *et al.* (2009). Gefitinib or Carboplatin/Paclitaxel in Pulmonary Adenocarcinoma. *New England Journal of Medicine*, **361**(10), 947–957.

Pál, D., Póczos, B., and Szepesvári, C. (2010). Estimation of rényi entropy and mutual information based on generalized nearest-neighbor graphs. In *Advances in Neural Information Processing Systems*, pages 1849–1857.

Reshef, D. N., Reshef, Y. A., Finucane, H. K., Grossman, S. R., McVean, G., Turnbaugh, P. J., Lander, E. S., Mitzenmacher, M., and Sabeti, P. C. (2011). Detecting novel associations in large data sets. *Science*, **334**(6062), 1518–1524.

Rubin, D. B. (1974). Estimating causal effects of treatments in randomized and nonrandomized studies. *Journal of educational Psychology*, **66**(5), 688–701.

Schäfer, J. and Strimmer, K. (2005). A shrinkage approach to large-scale covariance matrix estimation and implications for functional genomics. *Statistical applications in genetics and molecular biology*, **4**(1).

Scutari, M. and Brogini, A. (2012). Bayesian network structure learning with permutation tests. *Communications in Statistics-Theory and Methods*, **41**(16-17), 3233–3243.

Shannon, C. (1948). A mathematical theory of communication. *The Bell System Technical Journal*, **27**, 379–423.

Steuer, R. *et al.* (2002). The mutual information: detecting and evaluating dependencies between variables. *Bioinformatics*, **18**(Suppl 2), S231–S240.

Su, X., Zhou, T., Yan, X., Fan, J., and Yang, S. (2008). Interaction trees with censored survival data. *The International Journal of Biostatistics*, **4**(1).

Su, X., Tsai, C.-L., Wang, H., Nickerson, D. M., and Li, B. (2009). Subgroup analysis via recursive partitioning. *J. Machine Learning Research*, **10**, 141–158.

Tian, L., Alizadeh, A. A., Gentles, A. J., and Tibshirani, R. (2014). A simple method for estimating interactions between a treatment and a large number of covariates. *Journal of the American Statistical Association*, **109**(508), 1517–1532.

Tibshirani, R. (1996). Regression shrinkage and selection via the lasso. *Journal of the Royal Statistical Society. Series B (Methodological)*, pages 267–288.

Vieille, F. (2016). *aVirtualTwins: Adaptation of Virtual Twins Method from Jared Foster*. R package version 1.0.0.

Vinh, N. X., Epps, J., and Bailey, J. (2010). Information theoretic measures for clusterings comparison: Variants, properties, normalization and correction for chance. *Journal of Machine Learning Research*, **11**(Oct), 2837–2854.
